# Gene Expression-Based Classifiers Identify *Staphylococcus aureus* Infection in Mice and Humans

**DOI:** 10.1371/journal.pone.0048979

**Published:** 2013-01-09

**Authors:** Sun Hee Ahn, Ephraim L. Tsalik, Derek D. Cyr, Yurong Zhang, Jennifer C. van Velkinburgh, Raymond J. Langley, Seth W. Glickman, Charles B. Cairns, Aimee K. Zaas, Emanuel P. Rivers, Ronny M. Otero, Tim Veldman, Stephen F. Kingsmore, Joseph Lucas, Christopher W. Woods, Geoffrey S. Ginsburg, Vance G. Fowler

**Affiliations:** 1 Division of Infectious Diseases and International Health, Department of Medicine, Duke University, Durham, North Carolina, United States of America; 2 Section on Infectious Diseases, Durham Veteran’s Affairs Medical Center, Durham, North Carolina, United States of America; 3 Duke Institute for Genome Sciences and Policy, Duke University, Durham, North Carolina, United States of America; 4 van Velkinburgh Initiative for Collaborative BioMedical Research, Santa Fe, New Mexico, United States of America; 5 Immunology Division, Lovelace Respiratory Research Institute, Albuquerque, New Mexico, United States of America; 6 Department of Emergency Medicine, University of North Carolina School of Medicine, Chapel Hill, North Carolina, United States of America; 7 Department of Emergency Medicine, Henry Ford Hospital, Wayne State University, Detroit, Michigan, United States of America; 8 Center for Pediatric Genomic Medicine, Children’s Mercy Hospitals and Clinics, Kansas City, Missouri, United States of America; 9 Duke Clinical Research Institute, Durham, North Carolina, United States of America; National Institutes of Health, United States of America

## Abstract

*Staphylococcus aureus* causes a spectrum of human infection. Diagnostic delays and uncertainty lead to treatment delays and inappropriate antibiotic use. A growing literature suggests the host’s inflammatory response to the pathogen represents a potential tool to improve upon current diagnostics. The hypothesis of this study is that the host responds differently to *S. aureus* than to *E. coli* infection in a quantifiable way, providing a new diagnostic avenue. This study uses Bayesian sparse factor modeling and penalized binary regression to define peripheral blood gene-expression classifiers of murine and human *S. aureus* infection. The murine-derived classifier distinguished *S. aureus* infection from healthy controls and *Escherichia coli*-infected mice across a range of conditions (mouse and bacterial strain, time post infection) and was validated in outbred mice (AUC>0.97). A *S. aureus* classifier derived from a cohort of 94 human subjects distinguished *S. aureus* blood stream infection (BSI) from healthy subjects (AUC 0.99) and *E. coli* BSI (AUC 0.84). Murine and human responses to *S. aureus* infection share common biological pathways, allowing the murine model to classify *S. aureus* BSI in humans (AUC 0.84). Both murine and human *S. aureus* classifiers were validated in an independent human cohort (AUC 0.95 and 0.92, respectively). The approach described here lends insight into the conserved and disparate pathways utilized by mice and humans in response to these infections. Furthermore, this study advances our understanding of *S. aureus* infection; the host response to it; and identifies new diagnostic and therapeutic avenues.

## Introduction

Septicemia causes substantial morbidity and mortality among patients in the United States, with a rising burden of *Staphylococcus aureus* infection [1], [2]. Although blood cultures are the diagnostic gold standard for blood stream infection (BSI), sensitivity is limited and results are not rapidly available [3]. Such diagnostic delays can extend the time to administration of effective antibiotics, which is an independent risk factor for mortality [4], [5]. Conversely, diagnostic uncertainty also leads to high rates of empiric overtreatment, fueling the burden of antimicrobial resistance [6], [7]. Thus, novel approaches that are faster and more accurate are needed to differentiate between the major pathogens causing sepsis and BSI.

Whereas conventional diagnostic approaches have focused on identifying the infecting pathogen, a growing body of evidence suggests that the host’s inflammatory response to the pathogen also represents a potential diagnostic tool. *In vitro* and *in vivo* experiments have revealed fundamental differences in host response to Gram-positive and Gram-negative bacterial infection [8]–[10], including significant differences in Toll-like receptor (TLR) signaling [11], [12] and cytokine production [13], [14]. Distinctive gene expression profiles exist for viral [15], [16], bacterial [17], [18], and fungal infections [19], [20] in both animal model systems and *ex vivo* stimulation of human peripheral blood leukocytes. Peripheral blood mononuclear cell (PBMC) gene expression signatures have also been evaluated in humans for a variety of conditions including severe infection [21], bacterial vs. viral illness [10], systemic lupus erythematosus [22], atherosclerosis [23], and radiation exposure [24]. Taken together, these studies provide strong evidence that global changes in host blood gene expression patterns can be used to differentiate disease states.

The current study used *S. aureus* and *Escherichia coli* as prototypical Gram-positive and Gram-negative bacteria due to their prevalence and clinical relevance. Host gene expression was measured in mice with bacterial infection across multiple conditions. From these data, we derived a molecular classifier for *S. aureus* infection in inbred mice and validated it in a cohort of outbred mice. Next, we used host gene expression data from a well-characterized cohort of septic human subjects to identify a molecular classifier that accurately distinguished *S. aureus* BSI from *E. coli* BSI or uninfected controls. Murine and human *S. aureus* classifiers exhibited significant similarity particularly in comparing *S. aureus* infection to the healthy state. Furthermore, both murine and human classifiers were validated in an independent human cohort. This study is the first to demonstrate that the *in vivo* host response to Gram-positive infections is conserved from mouse to human and can be harnessed as a novel diagnostic strategy in patients with bacterial sepsis.

## Materials and Methods

### Ethics Statement

All animal experiments were carried out in strict accordance with the recommendations of NIH guidelines, the Animal Welfare Act, and US federal law. All animal procedures were approved by the Institutional Animal Care and Use Committee (IACUC) of Duke University (IACUC number: #1310905) which has been accredited by the Association for Assessment and Accreditation of Laboratory Animal Care (AAALAC) International. All animals were housed in a centralized and AAALAC accredited research animal facility that is fully staffed with trained husbandry, technical, and veterinary personnel. The Institutional Review Boards at Duke University Medical Center, the Durham VA Medical Center, and Henry Ford Hospital approved the human studies referenced in this work. Written informed consent was obtained for all subjects after the nature and possible consequences of the studies were explained.

### Preparation of Bacterial Cells

One methicillin-susceptible *S. aureus* (Sanger 476) and three methicillin-resistant *S. aureus* genetic backgrounds (USA100, USA300, and MW2) were used. Overnight *S. aureus* cultures were inoculated into fresh tryptic soy broth and incubated aerobically at 30°C to log-phase growth (optical density 600 nm of ∼1.0) [25]. Cells were harvested by centrifugation, rinsed, and resuspended in phosphate-buffered saline (PBS). *E. coli* O18:K1:H7 was grown at 30°C overnight in Luria-Bertani broth [26]. Cultures were then diluted with fresh medium and grown for an additional 1 to 2 hours. Upon reaching log phase, cells were treated as described for *S. aureus*.

### Human Subjects

Subjects were enrolled at Duke University Medical Center (DUMC; Durham, NC), Durham VAMC (Durham, NC), UNC Hospitals (Chapel Hill, NC), and Henry Ford Hospital (Detroit, Michigan) as part of a prospective, NIH-sponsored study to develop novel diagnostic tests for severe sepsis and community-acquired pneumonia (ClinicalTrials.gov NCT00258869) [27], [28]. Enrolled patients had a known or suspected infection and exhibited two or more Systemic Inflammatory Response Syndrome criteria [29]. Patients were excluded if they had an imminently terminal co-morbid condition, advanced AIDS (CD4 count <50), were being appropriately treated with an antibiotic pre-enrollment, or were enrolled in another clinical trial. Blood was drawn for microarray analysis on the day of hospital presentation with the exception of two subjects (S19 and S29). In these latter two cases, blood was not available for microarray preparation from that time point. However, blood drawn 24 hours into the hospitalization was available and so was used. Subjects in the current report had culture-confirmed monomicrobial BSI due to *S. aureus* (n = 32; median age 58 years; range 24–91) or *E. coli* (n = 19; median age 58; range 25–91). Uninfected controls (n = 43; median age 30 years; range 23–59) were enrolled at DUMC as part of a study on the effect of aspirin on platelet function among healthy volunteers [30]. Subjects were recruited through advertisements posted on the Duke campus. Blood used to derive gene expression data in these healthy controls was drawn prior to aspirin challenge.

### Murine Sepsis Experiments

Except where noted, mice were purchased from The Jackson Laboratory (Bar Harbor, ME) and allowed to acclimate for 7 days. All experiments were performed on 6–8 week old mice. For the murine *S. aureus* classifier, seven inbred mouse strains (3 mice/strain: 129S1/SvImJ, A/J, AKR/J, BALB/cByJ, C57BL/6J, C3H/HeJ, and NOD/LtJ) were IP inoculated with 10^7^ CFU/g of *S. aureus* Sanger476, euthanized at 2h after injection, and bled. This was repeated using the four different *S. aureus* genetic backgrounds (USA100, USA300, MW2, and Sanger476) in A/J mice (n = 3 per *S. aureus* background). For time series experiments, both A/J and C57BL/6J mouse strains were IP inoculated with *S. aureus* Sanger476 as above, and sacrificed at 2, 4, 6, and 12 h after injection (n = 5 per mouse strain at each time point). For survival experiments, mice were monitored twice daily after injection and culled upon reaching a moribund state. Animal sacrifice was carried out by carbon dioxide inhalation. Blood was collected by intracardiac puncture and stored in RNAlater at −70°C for microarray experiments.

The murine *E. coli* infection model was carried out as described above except a smaller inoculum (6×10^4^ CFU/g) was used. Furthermore, the time at which animals were sickest but still alive was 24 hours for *E. coli* inoculation, which is later than for *S. aureus*. Consequently, A/J and C57BL/6J mice inoculated with *E. coli* were sacrificed 24 h after challenge (n = 5 per mouse strain). Control mice were not injected.

Outbred CD-1 mice were purchased from Charles River Laboratories (Wilmington, MA) to validate the murine *S. aureus* classifier. CD-1 mice were IP inoculated with 10^7^ CFU/g of *S. aureus* (USA300 and Sanger 476) and 6×10^4^ CFU/g of *E. coli.* Animals including controls were sacrificed at 2 and 24 h post-infection (n = 10 mice per pathogen at each time point). Blood was collected and stored as described for the derivation cohort.

### Microarray Preparation (Additional Details Available in Methods S1)

Total RNA was extracted from mouse blood using the Mouse RiboPure Blood RNA kit (Ambion, Austin, TX) according to the manufacturer’s instructions. Globin mRNA was removed from whole blood RNA using the Globinclear kit (Ambion, Austin, TX). All samples passed the quality criteria of the Agilent Bioanalyzer and were used for microarray analysis. Since the total RNA yield of many samples was low, one round of linear amplification was performed for all samples using the MessageAmp Premier kit (Ambion, Austin, TX). RNA integrity numbers were calculated for all samples and found to be within tolerance limits. Microarrays were normalized using Robust Multichip Average (RMA). Affymetrix GeneChip Mouse Genome 430 2.0 Arrays were used (Santa Clara, CA). Biotin-labeled cDNA was hybridized to the arrays for 16 hours at 45°C according to the manufacturer’s instructions. Arrays were then washed and labeled with streptavidinphycoerythrin (strep-PE), and the signal was amplified using biotinylated antistreptavidin followed by another round of staining with strep-PE. These steps were performed on the Affymetrix fluidics station according to the recommended protocol. Amplification and microarray hybridization were performed at the Duke University Microarray Core. Labeled gene chips were scanned using an Affymetrix Genechip Scanner 7G (Santa Clara, CA). This array contains 45,101 probe sets to analyze the expression level of over 39,000 transcripts and variants from over 34,000 mouse genes.

Human microarrays were prepared by first extracting total RNA from human blood using the PAXgene Blood RNA Kit (Qiagen, Valencia, CA) according to the manufacturer’s recommended protocol including DNase treatment. RNA quantity and quality was assessed using the Agilent 2100 Bioanalyzer (Agilent, Santa Clara, CA). RNA integrity numbers were calculated for all samples and found to be within tolerance limits. Microarrays were normalized using RMA. Hybridization and microarray data collection was then performed at Expression Analysis (Durham, NC) using the GeneChip Human Genome U133A 2.0 Array (Affymetrix, Santa Clara, CA) according to the “Affymetrix Technical Manual”. Fluorescent images were detected in a GeneChip Scanner 3000 and expression data was extracted using the GeneChip Operating System v 1.1 (Affymetrix). All GeneChips were scaled to a median intensity setting of 500. Murine and human microarray data have been deposited in the NCBI GEO (accession # GSE33341).

### Deriving the Murine and Human *S. aureus* Classifiers

Microarray data was analyzed in two steps following the analysis strategy previously outlined and utilized [19]. First, a Bayesian sparse factor model was fit to the expression data without regard to phenotype [31], [32]. Second, factors were then used as independent variables to build a penalized binary regression with variable selection model [33] trained to identify *S. aureus* infection. In order to minimize issues with overfitting, batch was not included in the regression models. We used a Bayesian penalized regression technique for variable selection which allows for weighted model averaging of the resultant models, such that weights are computed from model fit on the training data [33]. The model averaging approach incorporates uncertainty in choice of model as well as regression coefficient. This has been shown to lead to out of sample predictive accuracies that are superior to penalized maximum likelihood approaches [34]. Assumptions for this approach are typical of probit regression including a linear response surface between predictors and the transformed latent probability variable. Genes were filtered for analysis using non-specific filtering for genes with high mean expression and high variance across samples. Samples with a high number of outlying genes were removed during the factor analysis. Mice were batched into discrete experiments with each experiment containing the relevant controls to avoid confounding. The development and application of this methodological approach has been previously described [15], [19], [31], [32], [35]–[42]. Using the same murine experimental data, another classifier was derived to classify methicillin-resistant vs. methicillin-sensitive *S. aureus* infection. The methodology was otherwise the same as that described above.

We fit a factor model on the human data independently from the mouse data. The factor model was fit to 9,109 genes after non-specific filtering to remove unexpressed and uniformly expressed genes. Z-scores were computed independently for each gene without regard to experimental design. Subjects with absolute z-scores greater than 3 in more than 5% of the genes on the array were identified as outliers and were not used to fit the factor model. The factor model was trained on the 91 samples (after removal of three outliers) from three batches of expression data, and this resulted in 79 factors. These 79 factors were then projected onto the full data set (including the three subjects removed for validation) with the goal of distinguishing *S. aureus* BSI from healthy controls or *E. coli* BSI. Leave-one-out cross-validation was utilized in order to control for overfitting of the penalized binary regression model. In order to minimize issues with overfitting, batch was not included in the regression models. Matlab (Natick, MA, USA) scripts to perform these operations are available. Nonparametric testing was used to evaluate model performance (Wilcoxon rank sum for 2-group comparisons or Kruskal-Wallis for 3 or more-group comparisons) unless otherwise indicated.

One limitation of this approach is that the marginal significance of genes within the factor-based classifier cannot be defined. Instead, gene lists were created to identify genes with differential expression between specified groups with respect to gene-level and factor-level analyses. For 3-group comparisons (*S. aureus* vs. *E. coli* vs. Healthy controls) one-way analysis of variance (ANOVA) was used. For pairwise comparisons, Student’s t-test was used. Results were statistically significant at p<0.05 after Bonferroni correction for multiple testing. Spreadsheets of gene/factor lists are provided as supplemental material.

### Creating a Human Ortholog of the Murine *S. aureus* Classifier

We used Chip Comparer (http://chipcomparer.genome.duke.edu/) to identify human orthologs for all possible mouse genes. When there were multiple orthologs, we preferentially used the anti-sense target probes that shared the fewest probes with other genes as identified by the probe label. Chip Comparer identified 17,600 probe sets on the Affymetrix GeneChip Human Genome U133A 2.0 Array that have orthologs in the Affymetrix GeneChip Mouse Genome 430 2.0 Array. Factor scores from the mouse factor model were estimated using this set of 17,600 genes as follows: Given a matrix of expression values, *X*, and a factor model *X = BF+e*, we first replaced missing values by mean expression levels for those genes. Step 2: Inverse regression was utilized to compute F*, to estimate the factor scores. Step 3: We estimated *X* by computing BF* and replaced missing values with the corresponding values from this matrix. Steps 2 and 3 were then repeated until the estimates for the missing values converged.

### External Validation in an Independent Cohort

To externally validate the murine and human *S. aureus* classifiers, we utilized publically available expression data from a pediatric cohort with *S. aureus* infection and healthy controls [18]. Hospitalized children with invasive *S. aureus* infection were enrolled with sample collection occurring after microbiological confirmation. Healthy controls included children undergoing elective surgical procedures and at healthy outpatient clinic visits. This dataset includes multiple expression platforms. For the purposes of consistency, we only included subjects with Affymetrix U133A data yielding 46 *S. aureus*-infected patients and 10 healthy controls. Given the absence of subjects with *E. coli* infection in the validation cohort, we derived new murine and human *S. aureus* classifiers that excluded animals or subjects with *E. coli* infection. These classifiers were derived and then projected onto the 56-sample validation cohort as described heretofore.

### Heat Map Generation

In order to generate heat maps of gene expression, we first turned to the factors from the murine and human *S. aureus* classifiers. Probes from each factor were identified and tested for differential expression in a one-way ANOVA. Probes with significantly different levels of expression after Bonferroni correction were retained. For the murine data, there were thousands of probes (∼1000–3000, typically) meeting these criteria. Consequently, the p-values were sorted in ascending order and the 100 most significant probes from each factor were retained. Duplicate probes across the factors were removed. The human expression heat map was created in the same manner except all significant probes are presented considering there were fewer factors and genes in the human *S. aureus* classifier as compared to the murine classifier. Heat maps were generated using Matlab (Natick, MA, USA).

### Pathway Analysis

Pathway analysis for functional annotation of genes was performed with the MetaCore tool of the GeneGO package (GeneGo, Inc., St. Joseph, MI, USA) (http://www.genego.com). P-values were assigned to pathways based on the number of genes mapping to a particular pathway relative to the total number of genes in that pathway. Statistically significant pathways were defined as a p-value <0.05 (False Discovery Rate [FDR]-adjusted) based on hypergeometric distributions [19].

## Results

### Murine Sepsis due to *S. aureus* and *E. coli*

Clinically relevant *S. aureus* infections in humans typically arise from a primary focus with secondary dissemination. To mimic this process, mice were inoculated via the intraperitoneal (IP) route [25]. Infection-susceptible and infection-resistant inbred mouse strains (A/J and C57BL/6J, respectively) [43], [44] were inoculated with *S. aureus* (Sanger476) or *E. coli* (O18:K1:H7) (n = 5 per mouse strain and bacterial species). A survival analysis was carried out to determine the optimal duration of infection for subsequent experiments (**Figure S1A**). Based on this data, A/J and C57BL/6J mice were infected with *S. aureus* (sacrificed at t = 0, 2, 4, 6, and 12 hours post-infection; n = 10 animals/time point) or *E. coli* (t = 0, 2, 6, 12, and 24 hours post-infection; n = 10 animals/time point). The effect of infection status, bacterial pathogen, and duration of infection on global patterns of gene expression was assessed using principal component analysis (PCA) (Partek Genomics Suite) (**Figure S1B-D**) [45]. Gene expression patterns clustered by infection status and by pathogen (*S. aureus* vs. *E. coli*). Animals infected with *S. aureus* demonstrated a time-dependent change in gene expression that first manifested at two hours, by which time bacteremia has occurred [46]. This pattern remained stable through 12 hours, when most animals have succumbed to sepsis. *E. coli*-infected animals did not reveal this time-dependent progression based on the time points sampled, but had a distinctly different pattern of gene expression that was evident at 2 hours and persisted through 24 hours following infection. A heat map depicting the time-dependent nature of these gene expression changes is presented in **Figure S2**.

### Peripheral Blood Gene Expression Signatures Classify *S. aureus*-infected from Uninfected Mice

To create a host gene expression-based classifier for *S. aureus* infection, mice from a variety of experimental conditions were utilized (n = 187 total). Seven strains of inbred mice were challenged with 4 *S. aureus* genetic backgrounds via IP inoculation and sacrificed at various time points as described in Experimental Procedures. The comparator group for model derivation included 50 A/J or C57BL/6J mice inoculated with *E. coli* (O18:K1:H7) as well as 54 non-inoculated mice. Whole blood mRNA was used to generate microarray expression data. A list of differentially expressed genes is presented in **Table S1**. **Figure S3** presents the number of overlapping genes in each pairwise comparison. Patterns of co-expressing genes were defined using sparse latent factor regression in an unsupervised manner (*i.e.* without knowledge of the source animal’s infection status) [31], [32]. Factor models are a well-known technique for describing correlation structure in high dimension, low sample size data sets. Our sparse latent factor model works by collecting genes that are highly correlated into groups. Predictive models are then built from the latent factors – vectors that describe the aggregate behavior of the group. Subsequently, these factors served as independent variables in a variable selection, binary regression model to distinguish animals with and without *S. aureus* infection. This approach was taken in lieu of using individual gene expression changes for several reasons. A given gene with biological relevance may be differentially expressed in response to *S. aureus* infection but not to the degree that would meet statistical significance. Considering this altered gene expression exists amid a network of other such changes, the collective perturbations in that particular pathway would be more easily detected using factor analysis. Furthermore, changes across multiple biological pathways will be reflected across multiple factors. These can then be collectively harnessed for their diagnostic potential using a binary regression model.

**Figure 1:**
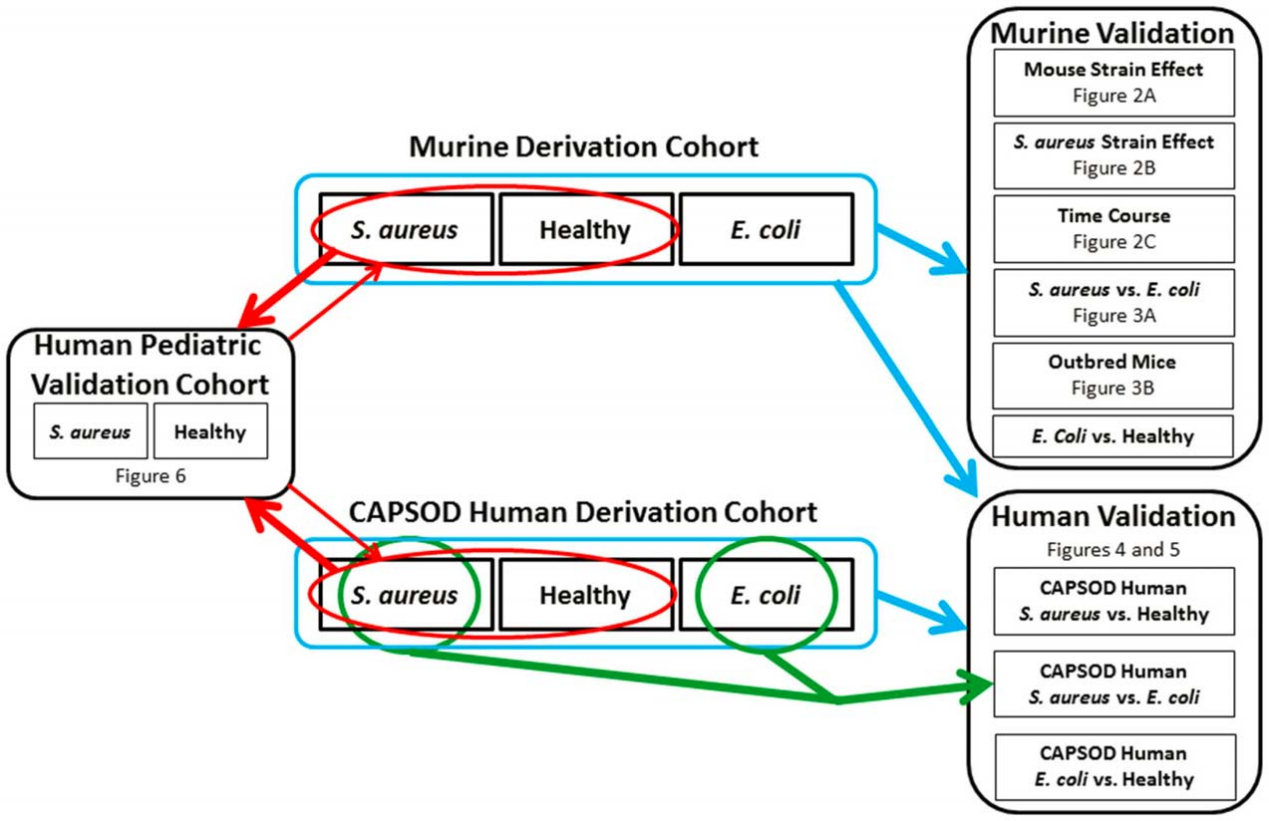
Schematic of derivation and validation cohorts. The Murine Derivation Cohort includes *S. aureus* infection (n = 83), healthy control mice (n = 54), and *E. coli* infection (n = 50). It served as a validation cohort to assess Mouse Strain Effect, *S. aureus* Genetic Background Effect, Time Course, and to compare *S. aureus* vs. *E. coli* and *E. coli* vs. Healthy. The murine *S. aureus* classifier was externally validated in Outbred Mice (n = 30) and the CAPSOD Human Cohort. The CAPSOD Human Cohort includes *S. aureus* BSI (n = 32), healthy volunteers (n = 43), and *E. coli* BSI (n = 19). It served as a validation cohort to compare *S. aureus* vs. Healthy, *S. aureus* vs. *E. coli*, and *E. coli* vs. Healthy. Model derivation and validation using the entire cohort of animals or humans is depicted by the blue outline and arrows. An independent classifier was generated using only subjects with *S. aureus* or *E. coli* BSI (green outline). This classifier was validated using leave one out cross validation (green arrow). The Human Pediatric Cohort (n = 46 *S. aureus*, 10 Healthy) used for external validation does not include patients with *E. coli* infection. Therefore, *S. aureus* classifiers were generated from the murine and CAPSOD cohorts that excluded *E. coli* data (red outline and thick red arrow). The Human Pediatric Cohort was used to derive a Human *S. aureus* vs. Healthy classifier which was validated in the *S. aureus*-infected and Healthy populations within the murine and CAPSOD human cohorts (thin red arrow).

Thirty factors were identified, of which 16 demonstrated a pattern of expression significantly associated with infection status (mFactors 15, 7, 23, 13, 9, 29, 28, 2, 17, 16, 21, 1, 5, 4, 26, and 19 in order of greatest significance; ANOVA; p<0.0017 for *S. aureus* vs. control vs. *E. coli* after Bonferroni correction; **Figure S4**). These 30 factors were fitted into a penalized binary regression model, termed the “murine *S. aureus* classifier”. The best performing model, as defined by the model with the largest log-likelihood value, included four factors (mFactors 7, 15, 23, and 26). Other models may be just as adequate, but we are only referring to this “top” model. Leave-one-out cross-validation was used to control overfitting and to estimate the model’s performance in subgroups of experimental conditions as described below (mouse strain, *S. aureus* genetic background, duration of infection, and bacterial species [*S. aureus* vs. *E. coli*]). A schematic of the derivation and validation experiments is depicted in **Figure 1**.

**Figure 2:**
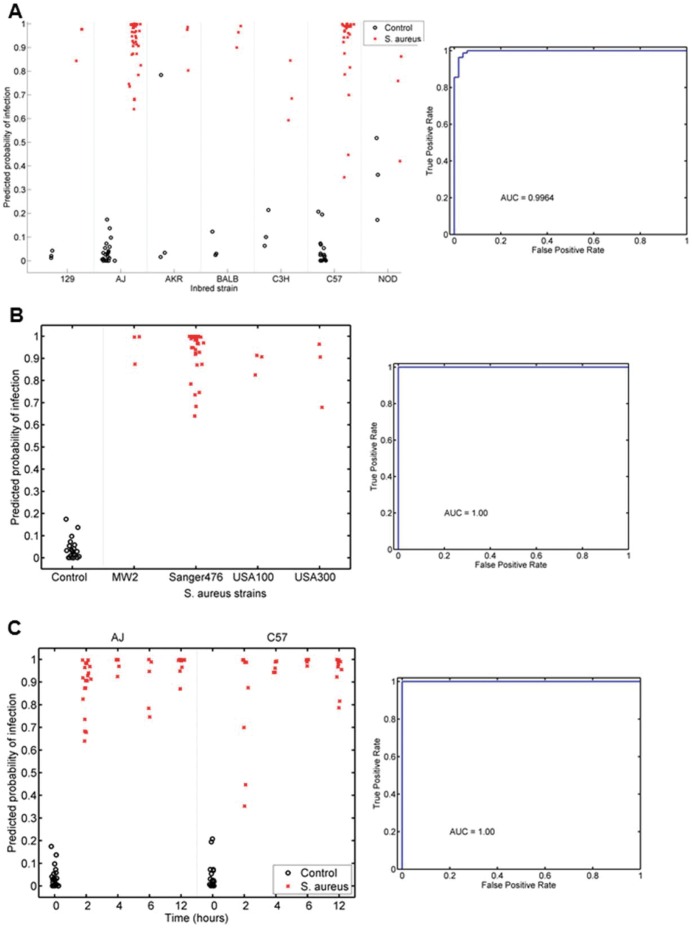
Murine *S. aureus* classifier accurately identifies *S. aureus* infection under a variety of conditions. Conditions represented include different murine hosts (A), bacterial genetic backgrounds (B), and time from inoculation (C). Animals with *S. aureus* infection are depicted by a red “x”. Uninfected control mice are depicted by black circles.

The ability of the murine-derived host gene expression classifier to identify *S. aureus* infection was tested in 7 inbred mouse strains of varying infection susceptibilities [43]. In all 7 strains, the murine *S. aureus* classifier accurately differentiated *S. aureus-*infected from control mice (p = 4.89×10^−16^; AUC = 0.9964) (**Figure 2A**). The ability to characterize *S. aureus* infection persisted when A/J mice (infection-susceptible) were challenged with four different *S. aureus* backgrounds: USA100 (the predominant US nosocomial methicillin resistant *S. aureus* [MRSA] genetic background); USA300 (the predominant US community-acquired MRSA genetic background); USA400 (MW2); and Sanger 476 (a methicillin-susceptible genetic background) (p = 1.92×10^−10^ vs. control mice; AUC = 1.00) (**Figure 2B**). Furthermore, the murine *S. aureus* classifier consistently discriminated *S. aureus* infected mice from controls at 2, 4, 6, and 12 hours post-inoculation (p = 4.41×10^−16^ vs. uninfected mice; AUC 1.00) (**Figure 2C** **)**. This time interval was selected because two hours is the earliest time point at which *S. aureus* can be cultured from blood; while 12 hours was the point at which animals began to die of *S. aureus* sepsis (**Figure S1A**). In summary, a classifier based on murine-derived host gene expression accurately identified the presence of *S. aureus* infection in mice under a variety of host, pathogen, and temporal conditions.

### Murine *S. aureus* Classifier Distinguishes *S. aureus*-infected from *E. coli*-infected Mice

**Figure 3:**
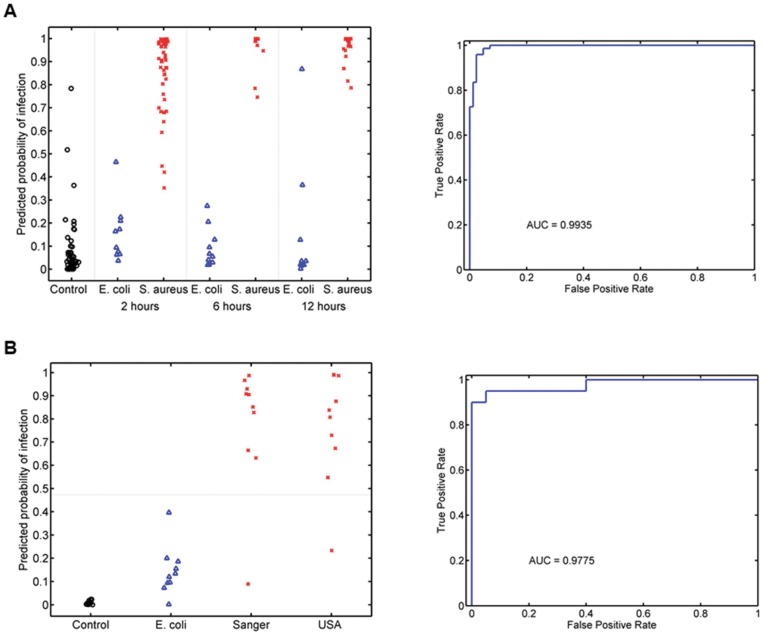
The murine *S. aureus* classifier differentiates *S. aureus* from *E. coli* infection. (A) Inbred mice were tested under three conditions: uninfected controls (black circles), *S. aureus* infected (red “x”), and *E. coli* infected (blue triangles). The y-axis represents the predicted probability that a given animal was infected with *S. aureus*. (B) The murine *S. aureus* classifier is validated in outbred CD-1 mice where it differentiates *S. aureus* infection from *E. coli* infection and uninfected controls.

Next, we determined whether the murine *S. aureus* classifier could differentiate *S. aureus* from *E. coli* infection. Both the infection-susceptible A/J and infection-resistant C57BL/6J strains were infected with either *S. aureus* (Sanger 476) or *E. coli* (O18:K1:H7). Animals were sacrificed at 2, 6, and 12 hours after inoculation. The murine *S. aureus* classifier correctly identified 50 of 53 (94.3%) animals as either infected with *S. aureus* or not at 2 hours (50/53), 100% of animals at 6 hours (n = 20), and 96.7% of animals at 12 hours (29/30) (**Figure 3A** **)**. This corresponded to an overall p-value of 7.94×10^−26^ by Kruskal-Wallis test (comparing *S. aureus* vs. *E. coli* vs. Healthy controls) with an AUC of 0.9935 across all time points. Next, the murine *S. aureus* classifier was independently validated in outbred CD-1 mice with *S. aureus* infection (Sanger 476 or USA300), *E. coli* infection (O18:K1:H7), or uninfected controls (10 animals per condition). The murine-derived *S. aureus* model accurately classified 95% of all animals where the reference standard was the known experimental condition (38/40; p = 1.47×10^−6^; 90% sensitivity and 100% specificity; AUC = 0.9775) (**Figure 3B**).

The murine *S. aureus* classifier was generated to identify *S. aureus* infection within a population including both healthy and *E. coli-*infected animals. However, it is possible this classifier is primarily distinguishing “sick” from “not-sick” phenotypes. In such a case, it would be expected that the classifier would still differentiate animals with *E. coli* infection from uninfected controls. However, this was not observed (AUC 0.5089; p = 0.8785) demonstrating the specificity of this classifier for *S. aureus* infection. Thus, a murine-derived host gene expression classifier accurately distinguished *S. aureus*-infected from *E. coli*-infected or uninfected mice across multiple host strains, pathogens, post-infection time points, and was validated in outbred mice.

Given this ability to discriminate infection due to different bacterial species, we further explored the potential for a factor-based classifier to distinguish infection due to methicillin-resistant (MRSA) or methicillin-sensitive *S. aureus* (MSSA), which have been shown to differ in their pathogenicity and virulence. The same 30 factors described above were fitted into a penalized binary regression model with the specific aim of differentiating MRSA from MSSA infection. Leave-one-out cross-validation was used to control overfitting and to estimate the model’s performance in a population of 19 MRSA-infected and 84 MSSA-infected mice (**Figure S5**). Despite some overlap, this classifier accurately differentiated infection due to MRSA or MSSA (AUC 0.8396; p = 4.14×10^−6^). Genes discriminating infection due to MRSA or MSSA that remained significant after adjusting for multiple tests are presented in **Table S2**.

### Human *S. aureus* Classifier

**Figure 4:**
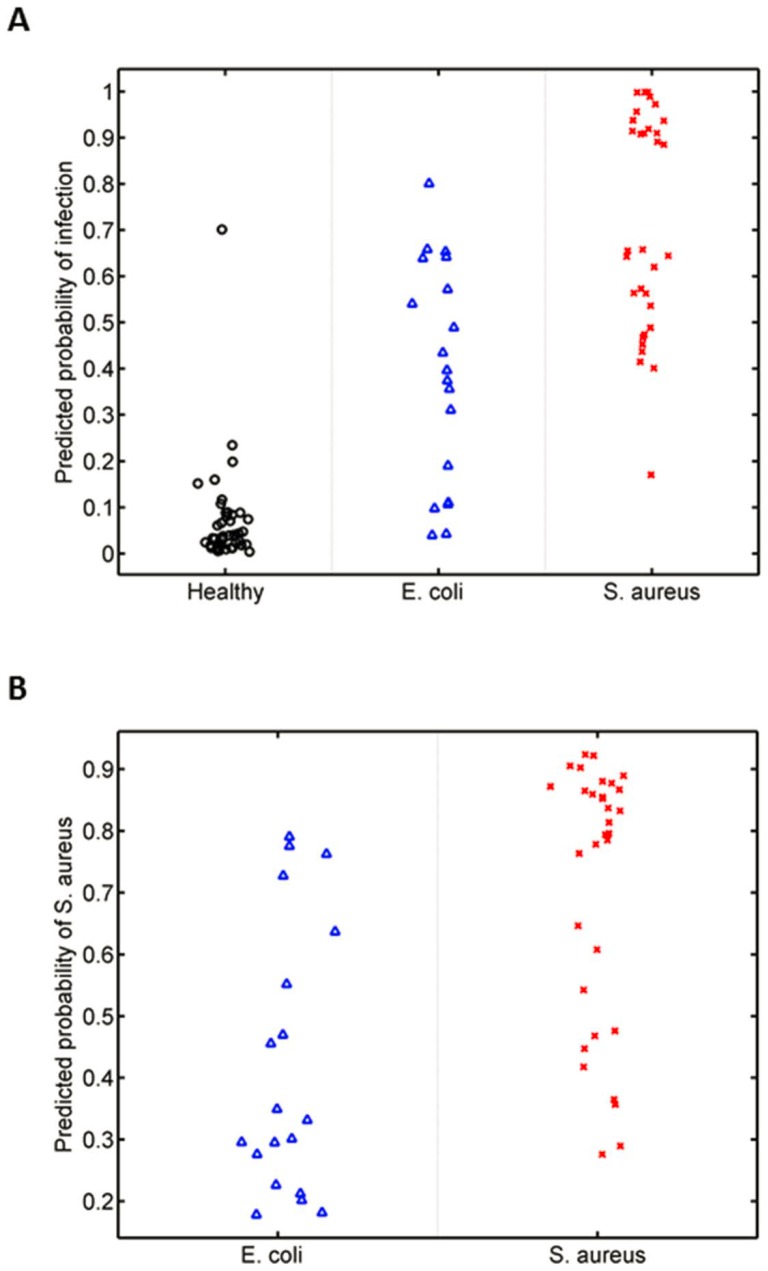
Performance of the human *S. aureus* classifier. (A) The human *S. aureus* classifier differentiates *S. aureus* BSI from both uninfected controls and *E. coli* BSI. (B) A separate classifier was generated using only *S. aureus* and *E. coli*-infected human subjects and tested using leave-one-out cross-validation.

We next determined whether peripheral blood gene expression in humans could yield a classifier for *S. aureus* BSI. Peripheral whole blood mRNA from 32 patients with *S. aureus* BSI, 19 patients with *E. coli* BSI, and 43 healthy control subjects were used to generate microarray data (**Table 1**). A list of differentially expressed genes is presented in **Table S3**. **Figure S6** presents the number of overlapping genes in each pairwise comparison. Seventy-nine factors were defined and fitted into a linear regression model trained to identify the presence of *S. aureus* BSI. Although 17 factors were independently associated with *S. aureus* BSI (**Figure S7**), only two factors remained in the best-performing model (hFactors 20 and 74). Similar to the murine *S. aureus* classifier, the human *S. aureus* classifier was generated blind to microbiological diagnosis in an unsupervised manner. Gender was controlled for in the model’s derivation considering the predilection for female sex in *E. coli* BSI (**Table 2**). We then estimated the model’s performance in phenotypic subgroups using leave-one-out cross-validation. The classifier accurately differentiated those with *S. aureus* BSI from healthy controls (72/75 correctly classified; AUC = 0.9898; p = 5.41×10^−13^) (**Figure 4A**). The human *S. aureus* classifier also correctly distinguished *S. aureus* from *E. coli* BSI in 82% (42/51) of cases (AUC = 0.8372; p = 6.77×10^−4^). When the human *S. aureus* classifier was applied to subjects with *E. coli* BSI vs. healthy controls, we observed an intermediate level of discrimination (56/62 correctly classified; AUC 0.9229; p = 1.38×10^−7^). This suggests that the human classifier is partially pathogen specific since *E. coli* BSI could also be distinguished from healthy controls but not with the same degree of accuracy as *S. aureus* BSI. A heat map depicting these gene expression changes is presented in **Figure S8**.

**Table 1:** Description of human subjects used to generate a *S. aureus* classifier.

Subject	Subject Category	Race	Age	Gender	Source of Infection	Positive Culture Sources	WBC	PMN %
S1	*S. aureus*	White	82	Male	Endocarditis	Blood	23.6	96.4
S2	*S. aureus*	White	70	Female	Skin	Blood, Wound, Operative cultures	11.6	N/A^a^
S3	*S. aureus*	Black	41	Male	Catheter^b^	Blood	14.8	N/A
S4	*S. aureus*	White	81	Male	Skin	Blood, Pleural fluid	16.2	N/A
S5	*S. aureus*	White	81	Male	Bone	Blood	17.3	N/A
S6	*S. aureus*	Black	55	Male	Catheter	Blood, Vascular catheter site	14.3	89.8
S7	*S. aureus*	Black	69	Female	Catheter	Blood	13	N/A
S8	*S. aureus*	Black	44	Female	Catheter	Blood	13	90
S9	*S. aureus*	Black	51	Male	Skin	Blood	6.9	73
S10	*S. aureus*	Black	47	Male	Skin	Blood	12	87
S11	*S. aureus*	White	36	Female	Endocarditis	Blood	22.7	85
S12	*S. aureus*	White	54	Male	Bone	Blood	9.8	88
S13	*S. aureus*	Black	55	Male	Bone	Blood, Skin, Synovial fluid	18.3	87
S14	*S. aureus*	Black	42	Male	Unknown	Blood, Urine, Sputum	7.6	82
S15	*S. aureus*	Black	52	Male	Bone	Blood	10.9	79
S16	*S. aureus*	Black	55	Male	Bone	Blood, Skin	28.6	95
S17	*S. aureus*	White	52	Male	Skin	Blood, Skin	17.9	84
S18	*S. aureus*	N/A	51	Female	Lung	Blood	19.8	78
S19 c	*S. aureus*	Black	40	Male	Skin	Blood, Skin	14.7	93
S20	*S. aureus*	White	60	Male	Skin	Blood	N/A	N/A
S21	*S. aureus*	Black	59	Male	Catheter	Blood	7.5	75.5
S22	*S. aureus*	Black	58	Male	Bone	Blood	27.9	N/A
S23	*S. aureus*	Black	77	Male	Urinary tract	Blood, Urine, Skin	9.8	80.3
S24	*S. aureus*	Black	91	Male	Bone	Blood	15.1	93
S25	*S. aureus*	White	75	Female	Catheter	Blood	6	92
S26	*S. aureus*	Black	58	Male	Catheter	Blood	23.9	87
S27	*S. aureus*	Black	24	Male	Urinary Tract	Blood, Urine, Sputum	16	76.4
S28	*S. aureus*	White	74	Male	Skin	Blood, Abscess	33.2	89
S29 c	*S. aureus*	Black	70	Male	Skin	Blood	19.6	82
S30	*S. aureus*	White	61	Male	Bone/CNS^d^	Blood, Abscess	10.4	86
S31	*S. aureus*, *S. pneumoniae*	Black	52	Male	Lung	Blood (*S. aureus*); Antigen test (*S. pneumoniae*)	6.1	93
S32	*S. aureus*	Black	38	Male	Endocarditis	Blood	16.8	94
E1	*E. coli*	Black	43	Female	Urinary tract	Blood, Urine	32.6	87.3
E2	*E. coli*	White	49	Female	Urinary tract	Blood	14	92.4
E3	*E. coli*	Black	44	Female	Urinary tract	Blood, Urine	15.7	N/A
E4	*E. coli*	White	70	Female	Urinary tract	Blood, Urine	20.7	88
E5	*E. coli*	Black	40	Male	Urinary tract	Blood, Urine	15	83
E6	*E. coli*	White	91	Female	Urinary tract	Blood, Urine	5.6	N/A
E7	*E. coli*	Black	25	Female	Urinary tract	Blood, Urine	11.1	88
E8	*E. coli*	White	62	Male	Urinary tract	Blood, Urine	13.3	N/A
E9	*E. coli*	Black	70	Male	Urinary tract	Blood	2.4	94
E10	*E. coli*	Black	32	Female	Urinary tract	Blood, Urine	25.1	N/A
E11	*E. coli*	White	54	Female	Urinary tract	Blood, Urine	10.8	90
E12	*E. coli*	White	74	Female	Urinary tract	Blood, Urine	7.3	97
E13	*E. coli*	Black	79	Female	Lung	Blood	16.9	77
E14	*E. coli*	Black	41	Male	Urinary tract	Blood, Urine	14.3	77.6
E15	*E. coli*	White	65	Male	Urinary tract	Blood, Urine	21.6	85
E16	*E. coli*	White	63	Female	Urinary tract	Blood, Urine	8.5	N/A
E17	*E. coli*	White	81	Female	Urinary tract	Blood, Urine	14.1	86.5
E18	*E. coli*	Black	69	Female	Urinary tract	Blood, Urine	11.1	67.6
E19	*E. coli*	White	55	Female	Urinary tract	Blood, Urine	7.2	87.5
H1	Healthy	Black	27	Male				
H2	Healthy	White	24	Female				
H3	Healthy	White	29	Female				
H4	Healthy	White	26	Male				
H5	Healthy	Asian	30	Male				
H6	Healthy	Black	24	Male				
H7	Healthy	White	N/A	Male				
H8	Healthy	Asian	24	Male				
H9	Healthy	Asian	23	Male				
H10	Healthy	White	50	Female				
H11	Healthy	White	23	Female				
H12	Healthy	White	24	Female				
H13	Healthy	White	44	Male				
H14	Healthy	White	24	Female				
H15	Healthy	White	28	Female				
H16	Healthy	White	26	Male				
H17	Healthy	Asian	30	Female				
H18	Healthy	Black	26	Male				
H19	Healthy	White	25	Male				
H20	Healthy	White	24	Male				
H21	Healthy	White	24	Male				
H22	Healthy	Asian	25	Female				
H23	Healthy	Black	24	Female				
H24	Healthy	White	43	Female				
H25	Healthy	White	26	Female				
H26	Healthy	Black	59	Male				
H27	Healthy	Black	25	Female				
H28	Healthy	White	24	Male				
H29	Healthy	White	25	Male				
H30	Healthy	White	26	Male				
H31	Healthy	White	24	Male				
H32	Healthy	White	26	Male				
H33	Healthy	N/A	25	Male				
H34	Healthy	White	53	Female				
H35	Healthy	Black	45	Female				
H36	Healthy	White	23	Male				
H37	Healthy	White	26	Female				
H38	Healthy	White	27	Male				
H39	Healthy	Asian	43	Female				
H40	Healthy	Black	32	Female				
H41	Healthy	N/A	25	Female				
H42	Healthy	Black	43	Female				
H43	Healthy	White	N/A	Female				

a N/A – Not available.

b Catheter refers to vascular catheters.

c Gene expression data for S19 and S29 was generated from blood drawn on the second hospital day. Blood drawn on the day of admission was otherwise used for all other infected subjects.

d This subject had vertebral osteomyelitis associated with an epidural abscess.

**Table 2:** Characteristics of human subjects used for *S. aureus* classifier derivation.

	*S. aureus* (n = 32)	Gram-negative (n = 19)	Healthy (n = 43)
Age in years, mean (range)	58 (24–91)	58 (25–91)	30 (23–59)
Gender, n (%)			
Female	6 (19)	14 (74)	21 (49)
Male	26 (81)	5 (26)	22 (51)
Race, n (%)			
Black	20 (63)	9 (47)	9 (21)
White	11 (34)	10 (53)	26 (60)
Asian	0	0	6 (14)
Unknown	1 (3)	0	2 (5)
Dialysis, n (%)	12 (38)	0	0
Diabetes, n (%)	13 (41)	3 (16)	0
Immunosuppression, n (%)	2 (6)	2 (11)	0

In the human *S. aureus* classifier described above, it is the inclusion of healthy controls that drives the discrimination from *S. aureus* BSI. Considering the clinical importance of differentiating Gram-positive from Gram-negative infections, rather than sick vs. healthy, we created a penalized binary regression model with the specific aim of differentiating human *S. aureus* (n = 32) from *E. coli* (n = 19) BSI. In this cohort, 52 factors were identified (different from the 79 factors identified when Healthy was included) of which only hFactor 40 remained in the top performing model after controlling for gender. Using leave-one-out cross-validation (**Figure 4B**), this model had a sensitivity of 62.5% (20/32 *S. aureus* BSIs correctly classified) but a specificity of 94.7% (18/19 *E. coli* BSIs correctly classified). This corresponds to an AUC of 0.8503 (p = 3.47×10^−5^).

### A Murine *S. aureus* Classifier Identifies *S. aureus* Infection in Humans

**Figure 5:**
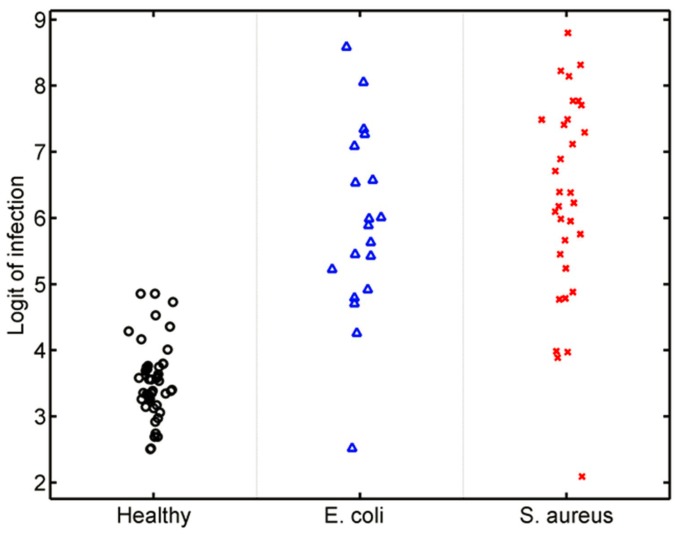
Projecting the mouse *S. aureus* classifier onto human subjects. The murine *S. aureus* classifier identifies humans with *S. aureus* BSI, but does not differentiate *S. aureus* from *E. coli* BSI.

We then determined whether the murine *S. aureus* classifier could identify *S. aureus* BSI in humans. To accomplish this, the murine *S. aureus* classifier was projected onto human gene expression data. Specifically, Chip Comparer (http://chipcomparer.genome.duke.edu/) provided a modified representation of the Affymetrix Mouse Genome 430 2.0 Array that only included orthologs of transcripts represented on the Affymetrix Human Genome U133A 2.0 Array. This resulted in a murine *S. aureus* classifier consisting only of genes with human orthologs (68.6% of the total array representation). We then evaluated this classifier in our human cohort. To account for potential species-specific variation in gene expression, predicted probabilities were plotted on a logit rather than a probabilistic scale. Using this murine *S. aureus* classifier, human patients with *S. aureus* BSI were distinguished from healthy controls (AUC = 0.9484; p = 4.00×10^−11^) (**Figure 5**). Thus, the host response to *S. aureus* infection was sufficiently conserved that a predictive model generated in one species (*Mus musculus*) identified *S. aureus* BSI in another (*Homo sapiens*). However, the murine-derived *S. aureus* classifier did not differentiate between *S. aureus* and *E. coli* BSI in humans (AUC = 0.5905; p = 0.2883).

### Validation of Murine and Human Classifiers in an Independent Pediatric Population

**Figure 6:**
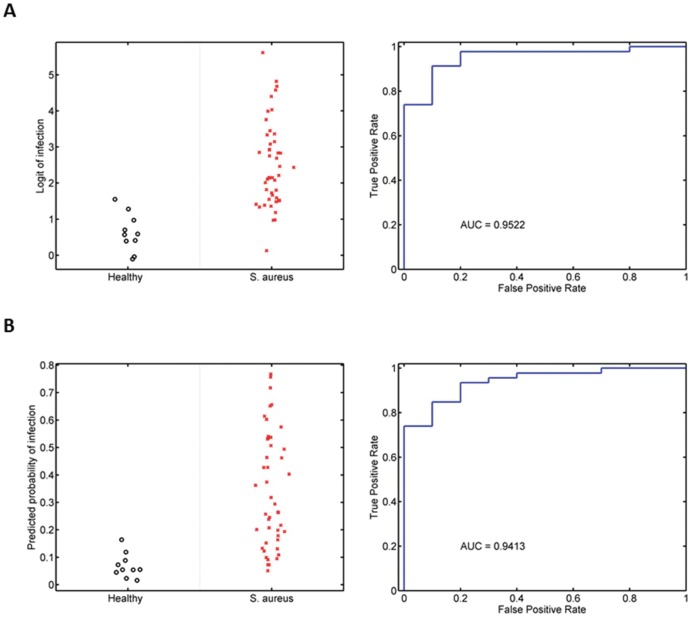
Validation in an independent human cohort [18]. (A) The murine *S. aureus* classifier differentiates between *S. aureus* infection and healthy. (B) The human *S. aureus* classifier differentiates between *S. aureus* infection and healthy.

We externally validated the murine and human *S. aureus* classifiers in an independent human cohort [18]. This validation cohort consisted of pediatric patients hospitalized due to invasive *S. aureus* infection (n = 46) and healthy controls (n = 10) who had gene expression data generated on a compatible platform (U133A array) with that used in this study. This cohort did not enroll children with *E. coli* infections. For this reason, we excluded *E. coli* infection from both classifiers. New murine and human *S. aureus* classifiers were developed in the same manner described above but without *E. coli*-related expression data. This modified murine *S. aureus* classifier was comprised of mFactors 7, 15, and 26 but not mFactor23. The modified human *S. aureus* classifier only contained hFactor4. Both the murine and human *S. aureus* classifiers differentiated children with *S. aureus* infection from healthy controls in this validation cohort (murine classifier AUC = 0.9522, p-value = 9.03×10^−6^ [**Figure 6A**]; human classifier AUC 0.9217, p-value 3.48×10^−5^ [**Figure 6B**]). The converse was also true. A *S. aureus* classifier trained on this independent pediatric cohort accurately discriminated *S. aureus* infection from healthy controls in our CAPSOD human cohort (70/75 correctly classified; AUC = 0.9775, p-value = 2.03×10^−12^) and murine cohort (123/137 correctly classified; AUC = 0.9255; p = 4.56×10^−17^).

### *S. aureus* Infection Induces Similar Host Gene-expression Responses in Mouse and Human

Pairwise comparisons were performed to identify genes with significantly different levels of expression (after Bonferroni correction). Comparisons included *S. aureus* infection vs. Healthy, *E. coli* infection vs. Healthy, and *S. aureus* vs. *E. coli* infection in mice and humans (**Tables S1 and S3**). Genes from each pairing were entered into the GeneGo pathway map database. The 50 most significant biological pathways arising from the pairwise comparisons are presented in **Table S4**. The genes represented within common pathways are presented in **Table S5**. A similar number of pathways overlapped between the murine and human responses to *S. aureus* (12 of the top 50) and *E. coli* (14 of the top 50) infection. Most of the overlapping pathways in the murine and human responses to both *S. aureus* and *E. coli* belonged to the broad category of immune response including CD28, ICOS, and the MEF2 pathway. Cytoskeletal remodeling (TGF and WNT) and apoptosis were also common to both infection types in mice and humans. Some pathways were highly significant in the *S. aureus* vs. Healthy comparison but not manifest in *E. coli* vs. Healthy such as NF-κB-associated pathways; the CD40 immune response pathway; and clathrin-coated vesicle transport. As expected, these pathways were also differentially manifest in the direct comparison of murine *S. aureus* and *E. coli* infection. We did not identify any statistically significant probes that distinguished human *S. aureus* from *E. coli* BSI. One probe, corresponding to the F2RL3 gene, nearly met this statistical cutoff (p-value 5.94×10^−6^ with a cutoff of 2.24×10^−6^). F2RL3 encodes proteinase-activated receptor 4 [47]. This molecule is a G-protein coupled receptor activated by thrombin and trypsin but has not previously been implicated in the sepsis or immune response. It is expressed in multiple tissues with high levels in the lung, pancreas, thyroid, testis, and small intestine but not peripheral blood or lymphoid tissues [47].

## Discussion

Early diagnostic strategies for *S. aureus* BSI could improve patient care by reducing the time required to establish the diagnosis and provide appropriate treatment while avoiding unnecessary anti-MRSA antibiotics. The current investigation contributes to this goal through three key findings. First, *S. aureus* infection induces conserved host gene expression responses in mice that can differentiate from *E. coli*-infected or uninfected mice. This discovery was consistent and robust across multiple inbred mouse strains, *S. aureus* genetic backgrounds, time points, and was validated in outbred mice. The validation step strengthens generalizability and is an important improvement over previous murine gene-expression based classifiers that were developed and tested in only a single inbred mouse strain including the fields of infectious diseases [19], [48], [49]; cancer progression [50], [51]; and aging [52], [53]. Furthermore, this murine predictor was specific for *S. aureus* infection and not simply a marker of illness based on the observation that mice with *E. coli* sepsis could not be distinguished from healthy, uninfected animals. The murine *S. aureus* classifier performed equally well at multiple time points despite progression of illness lending additional support to the specificity of this classifier. Second, human-derived host gene expression signatures differentiated *S. aureus* BSI from *E. coli* BSI or uninfected controls. In contrast to the murine-based classifier, the human-based model was less pathogen specific but still provided a significant degree of differentiation between *S. aureus* and *E. coli* BSI. Finally, the responses to *S. aureus* infection are highly conserved at the transcriptional and pathway level. This conserved response allowed us to validate the murine- and human-derived *S. aureus* classifiers in an independent cohort of *S. aureus*-infected patients.

Previous efforts to identify a discriminatory host gene expression signature for Gram-positive versus Gram-negative infections have yielded inconsistent results. This is likely due to the observation that transcriptional data derived from complex phenotypes such as infection do not produce just one predictive gene set, but rather generate multiple gene sets associated with the phenotype in question [54]. Some studies report a common pattern of host gene expression [55]–[57], whereas others have identified different expression profiles [8]–[10], [58]. In the current investigation, we utilized well-established methodologies [15], [19], [31], [36], [38]–[41] to derive predictors for *S. aureus* infection in both mice and humans from gene expression data. A key component of this methodology was a dimensional reduction step generating sets of co-expressed genes, termed “factors”. We observed that multiple, individual factors differentiated between various infection states although none performed universally well. For example, mFactor15 was associated with the lowest overall p-value during model generation. The AUC was 0.9587 for *S. aureus* vs. uninfected control mice but only 0.7942 for *S. aureus* vs. *E. coli*. In contrast, mFactor23 had an AUC of 0.9800 for *S. aureus* vs. *E. coli* but an AUC of 0.5926 for *S. aureus* vs. uninfected control mice. In order to generate a more robust classifier, factors were used as independent variables to generate a binary regression model. Factor models are an excellent technique for estimating correlation structure in very high dimensional data sets. This comprised the second step in generating the *S. aureus* predictors. It was only by including all factors to build the classifier that we could validate the model in the broadest set of conditions including different bacterial pathogens. However, because factors are typically made up of many genes, it is difficult to estimate the marginal effect of removing single genes from predictors. As such, it can be challenging to move from predictors based on factors to predictors based on small gene subsets. Although redundancy among the genes in a molecular classifier is expected and is a potential limitation, such redundancy can also improve robustness for a specific phenotype [54] as is likely to be the case in discriminating *S. aureus* from *E. coli* infection in mice. Comparisons at the individual gene level, as with pairwise comparisons, are likely to reveal differences in relatively simple biological responses. In contrast, dimension reduction with factor modeling as utilized in this study incorporates differences across multiple pathways, allowing for the detection of changes in a more complex pathobiology. Additionally, our factor model construction does not incorporate known biological pathways. This leads to gene groupings that are sometimes difficult to interpret. The advantage of the approach is the extreme dimension reduction which allows for discovery and cross-validation on very small data sets. This is one possible explanation for why the human *S. aureus* classifier differentiated *S. aureus* from *E. coli* whereas no genes met the threshold for differential expression after Bonferroni correction in a pairwise comparison between these two patient populations. The strength of this approach is offset by the possibility that smaller or transient changes in gene expression might be missed. It should be noted that the classifiers described in this study are not intended to be of clinical grade, which would require a more restrictive set of discriminating genes. Furthermore, there are likely many combinations of genes and factors that would perform similarly to that described here. This study presents findings related to the best performing classifier using the described methodologies. Defining the smallest, non-redundant set of genes that retains adequate discriminating power would be a vital next step in generating a clinically-useful diagnostic. In addition, any host response-based diagnostic requires validation across a range of clinical states. Immunocompromise is a particular condition in which it cannot be assumed the host immune response follows the paradigms identified here.

The murine model has been effectively used to gain insights into the pathophysiology of sepsis in general and *S. aureus* in particular [43], [44]. Murine-derived gene expression signatures have also been successfully translated to non-infectious human conditions such as radiation exposure and breast cancer [24], [59], [60]. Here, we describe the robust performance of a murine-derived *S. aureus* classifier in both mice and humans and also offer several lines of evidence supporting a partially conserved host response to *S. aureus* infection in both host species. First, the murine-based predictor could differentiate human *S. aureus* BSI from uninfected controls. Second, the genetic pathways were highly conserved. For example, most of the relevant murine pathways were also significantly associated with *S. aureus* BSI in humans. Finally, the murine-based predictor was highly accurate in classifying *S. aureus* infection in an independent human cohort.

Despite the robust performance of the murine classifier when applied to a human population, the ideal animal model for human sepsis remains elusive [61]–[63]. For example, virtually no murine-based sepsis studies have been replicated in patients [64], [65]. Other sepsis studies in mice and humans yield discordant results. For example, the impact of TNF-α receptor therapy on septic mice [66] and humans [67] yielded contradictory results. In fact, more than 60 incongruities between murine and human immune systems have been recognized many of which involve host-pathogen interactions [68], [69]. Our results are consistent with these earlier observations. For example, we encountered inconsistences between murine and human responses to *S. aureus* such that a minority (12) of the top 50 pathways overlapped between the two species. Moreover, the human response to *S. aureus* when compared to *E. coli* was differentiated by only one gene, F2RL3, which nearly reached the threshold for statistical significance. This is in contrast to the many genes identified differentiating the murine response to *S. aureus* and *E. coli* infection although F2RL3 is notably absent from this list. These host species-specific differences in sepsis, as well as infection-specific characteristics such as anatomic site of infection (e.g. genitourinary tract for *E. coli* vs. skin/soft tissue for *S. aureus*) limit our ability to apply knowledge gained from animal sepsis models to humans. It is also worth noting that batch effects and their correction may introduce bias in the form of false positives in the gene selection output [54]. However, this effect would be equally distributed among the *S. aureus* infected, *E. coli* infected, and healthy subjects. Finally, the ability to distinguish bacterial sepsis from healthy is expectedly easier than the finer distinction between two offending bacterial pathogens. It is therefore not surprising that the murine *S. aureus* predictor did not differentiate *S. aureus* from *E. coli* infection in the human cohort. Comorbid disease such as diabetes or end-stage renal failure, which we observed in a minority of the infected human cohort, could be confounding the analysis and driving the differentiation between healthy human controls and those with infection (*S. aureus* or *E. coli* BSI). Without controlling for comorbid disease, such a confounding effect cannot be excluded. However, the human *S. aureus* classifier performed exceptionally well in differentiating infected individuals from healthy controls even in those patients without comorbid disease. Furthermore, the murine classifier (derived from mice without comorbid disease) could still differentiate infected human subjects from the healthy human cohort. These factors make it unlikely that comorbid disease is playing a significant role in the analysis although future attempts at deriving a gene-expression-based classifier should make accommodations for the possible confounding effect of comorbid disease.

Gene expression changes in peripheral blood cells drive the derivation of both the murine and human *S. aureus* classifiers. It is conceivable these gene expression changes are reflective of transcript abundance driven by myeloid cell lineage expansions and are not pathogen or infection specific. However, previously published data and work presented here suggest this is not the case. For example, Ardura et al. found no differences in the absolute numbers of total B and T cells in patients with *S. aureus* infection compared to healthy controls [18]. Yet the abundance of lymphocyte-specific transcripts was significantly reduced. In contrast, expansion of the myeloid lineage was associated with high levels of expression among genes associated with neutrophil function. A similar independence between lymphocyte counts and differential gene expression within this lineage was observed in an independent pediatric sepsis cohort [70]. In another example, transcript abundance due to cell lineage expansions was not the primary factor in the development of a tuberculosis-specific gene expression signature [71]. Rather, it is changes in cellular composition and altered gene expression that drive such signatures. The data presented here also indicates that the *S. aureus* classifiers are not being driven by lineage-specific transcript abundance. Specifically, the total leukocyte count and cell lineage distribution (based on routine automated differential measurements) were not different between patients with *S. aureus* infection and *E. coli* infection (15.7×10^9^/L with 86.2% neutrophils vs. 14.1×10^9^/L with 85.8% neutrophils, respectively). However, the human *S. aureus* classifier was still able to differentiate infection due to the two pathogens. The murine *S. aureus* classifier was highly successful in differentiating *S. aureus* infection from healthy and from *E. coli* infection yet was unable to differentiate *E. coli* from healthy. This result would not be expected if transcript abundance was driving the derivation of the classifier.

The overlap we observed in the gene expression response to *S. aureus* infection in mouse and human was also consistent with published studies. NF-κB signaling pathways have been identified as a critical component of the murine response to infection [72], which was mirrored in the murine and human data presented here. Similar gene expression-based analyses of the human response to bacterial infection have also revealed the importance of other biological pathways including MHC class I and II antigen presentation, immunological synapse formation, TGF-β receptor signaling, TGF and WNT-dependent cytoskeleton remodeling, and T-cell receptor signaling [10], [18], [73], all of which were significantly associated with *S. aureus* infection in this study. Hence, mice and humans utilize many of the same or overlapping pathways in response to bacterial sepsis supporting the potential utility of murine-based diagnostics for human disease.

*S. aureus* continues to evolve as a pathogen and leads to a disproportionate burden of sepsis morbidity and mortality. This study takes significant steps forward on multiple levels in the ongoing effort to understand this pathogen; the host response to it; and identify new diagnostic and therapeutic avenues. We describe a potential diagnostic modality capable of differentiating infection from health across species. More importantly, host gene expression classifiers can differentiate infection due to *S. aureus* from that of *E. coli* but this effect is less pronounced in the complex human host. The approach described here also affords great insight into the conserved and disparate pathways utilized by mice and humans in response to these infections. Not only have we provided evidence to support the paradigm shift in how we think about diagnostics, but we have also identified new areas for research into the pathways that subserve sepsis pathophysiology.

## Supporting Information

Figure S1 **Bacterial challenge experiments.** (A) Survival curves for A/J and C57BL/6J mice following an intra-peritoneal infection with *S. aureus* (1×10^7^ CFU/g) or *E. coli* (6×10^4^ CFU/g). Principal Components Analysis plots of the samples in the dataset. Samples are colored by infection status and pathogen. (B) *S. aureus* infection by time after inoculation (n = 10 animals/time point). (C) *E. coli* infection by time after inoculation (n = 10 animals/time point). (D) PCA differentiated by pathogen.
(DOC)

Figure S2 **Heat maps of genes contributing to the murine** ***S. aureus*** **classifier.** (A) Genes within the top five factors contributing to the murine *S. aureus* classifier were identified and ranked by p-value after Bonferroni correction. A subset of genes (393 after removing duplicates) is depicted here, stratified by pathogen. (B) The same genes depicted in part (A) are categorized first pathogen and then by time since infection.
(DOC)

Figure S3 **Venn diagram demonstrating the number of overlapping probes in each murine experimental group pairwise comparison.** Probes were included that had significantly different levels of expression after Bonferroni correction.
(DOC)

Figure S4 **Sixteen murine factors independently associated with** ***S. aureus*** **infection projected onto healthy controls (left panel, black circles), animals with** ***E. coli*** **infection (middle panel, blue triangles), and animals with** ***S. aureus*** **infection (right panel, red “x”).** The y-axis represents the factor score.
(DOC)

Figure S5 **A factor-based classifier distinguishes MRSA from MSSA infection in mice. An ROC curve is shown for this classification.**
(DOC)

FIgure S6 **Venn diagram demonstrating the number of overlapping probes in each human experimental group pairwise comparison.** Probes were included that had significantly different levels of expression after Bonferroni correction. No probes met this cutoff for the *S. aureus* vs. *E. coli* comparison.
(DOC)

Figure S7 **Seventeen human factors independently associated with** ***S. aureus*** **BSI projected onto healthy controls (left panel, black circles), subjects with** ***E. coli*** **BSI (middle panel, blue triangles), and subjects with** ***S. aureus*** **BSI (right panel, red “x”).** The y-axis represents the factor score.
(DOC)

Figure S8 **Heat map of genes contributing to the human** ***S. aureus*** **classifier.** Genes within the top two factors contributing to the human *S. aureus* classifier were identified and ranked by p-value after Bonferroni correction. A subset of genes (86 after removing duplicates) is depicted here, stratified by pathogen.
(DOC)

Table S1 **Probes, ranked by p-value, that were differentially expressed (after Bonferroni correction) in mice with** ***S. aureus*** **infection vs. Healthy controls;** ***S. aureus*** **vs.** ***E. coli*** **infection; and** ***E. coli*** **vs. Healthy controls.** Also presented is the average probe expression in each comparator group and the fold-change within the pairwise comparison.
(XLSX)

Table S2 **Probes and corresponding genes that were differentially expressed (after Bonferroni correction) in mice with MRSA vs. MSSA infection.**
(DOC)

Table S3 **Probes, ranked by p-value, that were differentially expressed (after Bonferroni correction) in humans with** ***S. aureus*** **infection vs. Healthy controls;** ***S. aureus*** **vs.** ***E. coli*** **infection; and** ***E. coli*** **vs. Healthy controls.** Also presented is the average probe expression in each comparator group and the fold-change within the pairwise comparison.
(XLSX)

Table S4 **Pathway analysis for the genes from pairwise comparisons in the mouse and human study.** Top 50 ranked pathways from GeneGo MetaCore pathway analysis based upon p-value. Shaded text corresponds to pathways that are present in both the mouse and human response to the specified pathogen.
(XLSX)

Table S5 **Genes in pathways common to murine and human responses to infection.** Human genes are in the shaded cells. Murine genes are in the unshaded cells.
(XLSX)

Methods S1 **A detailed description of microarray preparation.**
(DOC)
