# Supplementary figures and images for: Gene Expression-Based Classifiers Identify Staphylococcus aureus Infection in Mice and Humans
Source: PLoS One. 2013 Jan 9;8(1):e48979. doi: 10.1371/journal.pone.0048979 (PMC3541361; doi:10.1371/journal.pone.0048979)

**Figure S1**


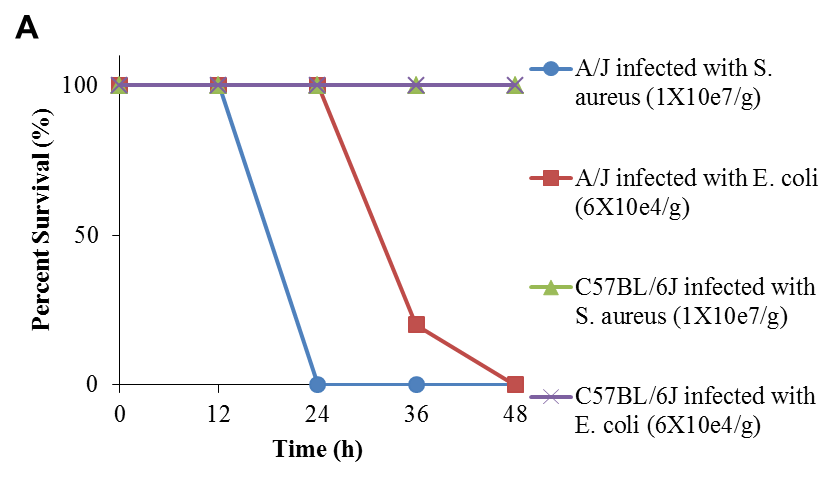


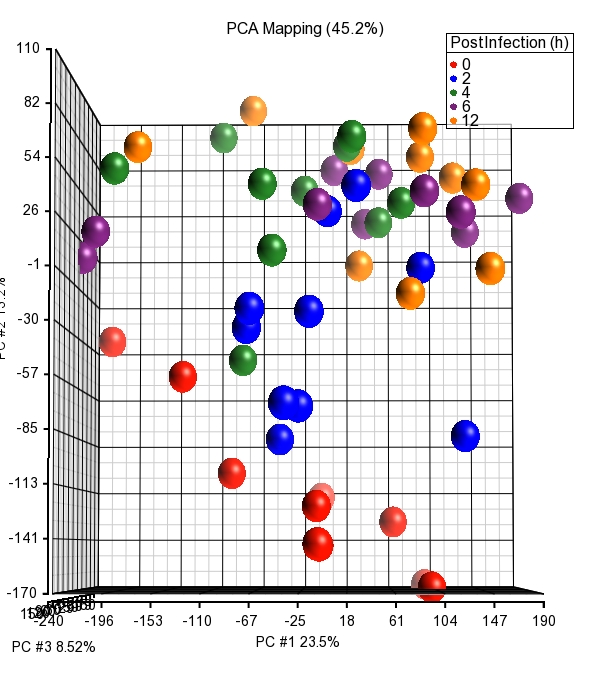


Uninfected

2h

4h

6h

12h

**B**


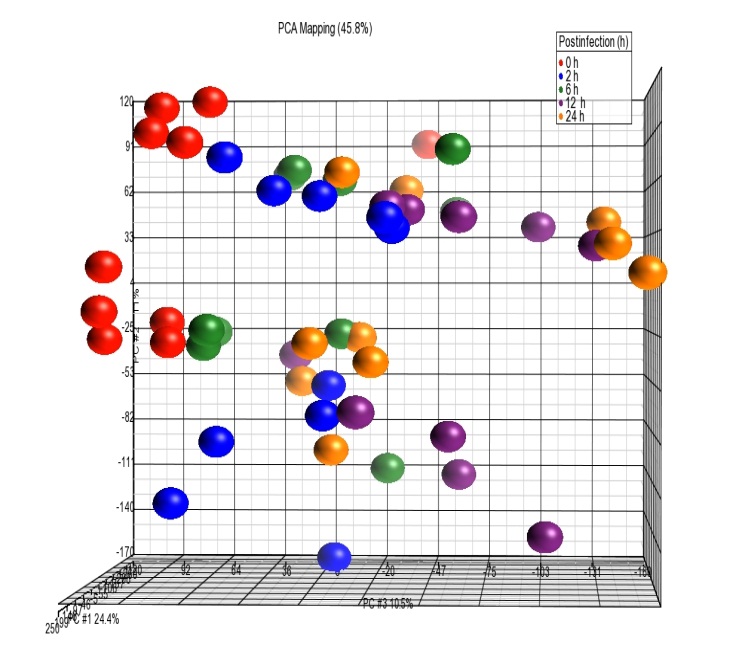


Uninfected

2h

6h

12h

24h

**C**


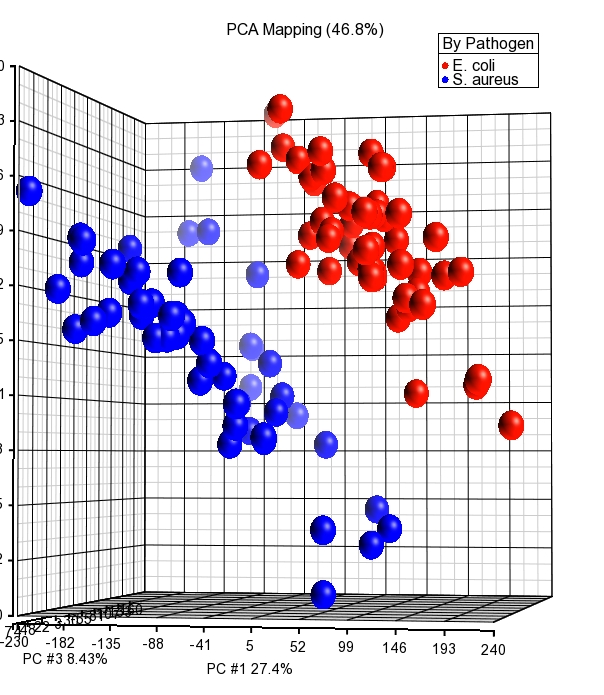


*E. coli*

*S. aureus*

**D**

Supplement: Figure S1 — Bacterial challenge experiments. (A) Survival curves for A/J and C57BL/6J mice following an intra-peritoneal infection with S. aureus (1×10^7^ CFU/g) or E. coli (6×10^4^ CFU/g). Principal Components Analysis plots of the samples in the dataset. Samples are colored by infection status and pathogen. (B) S. aureus infection by time after inoculation (n = 10 animals/time point). (C) E. coli infection by time after inoculation (n = 10 animals/time point). (D) PCA differentiated by pathogen. (DOC) [file pone.0048979.s001.doc]

**Figure S2**


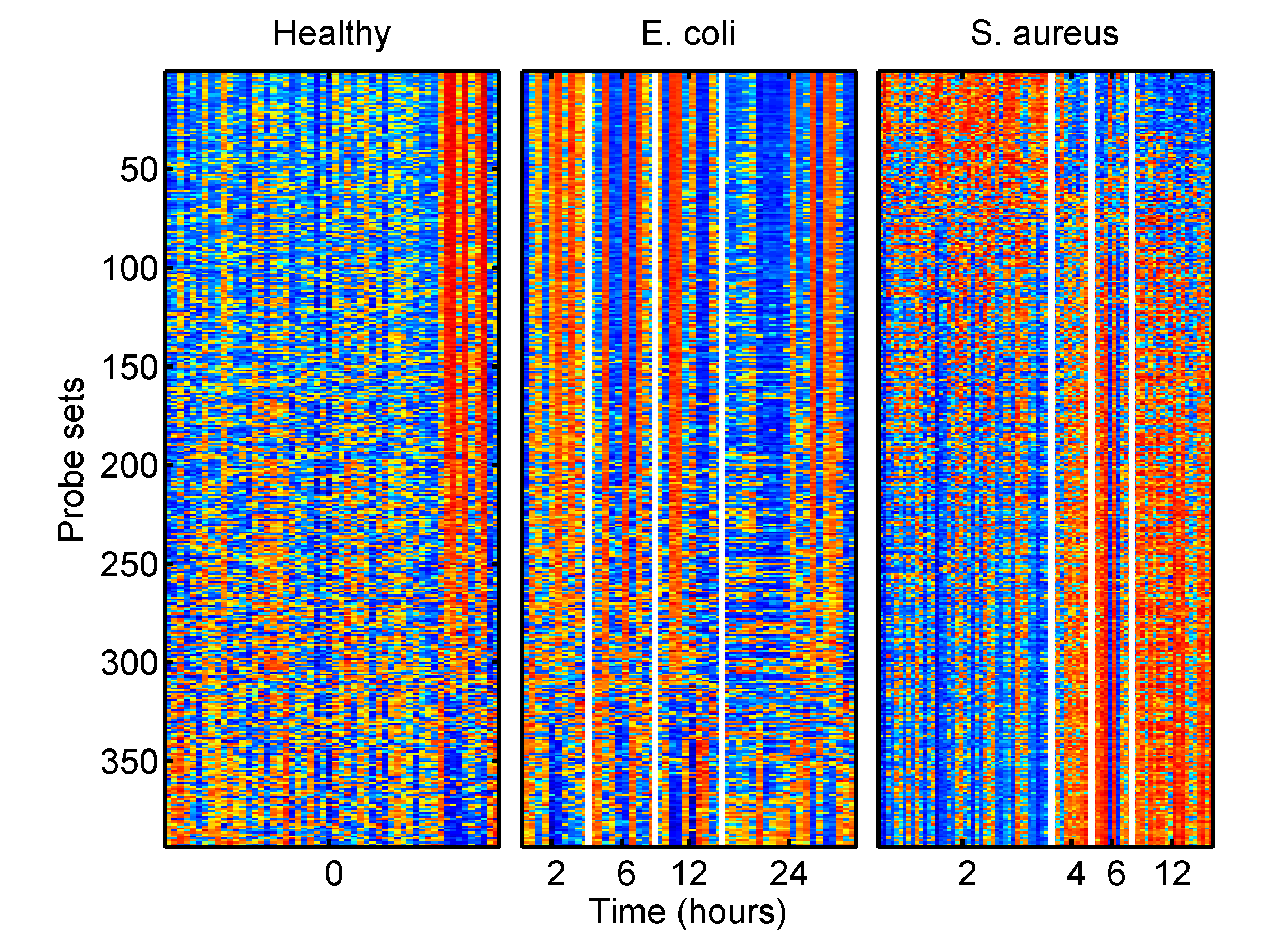

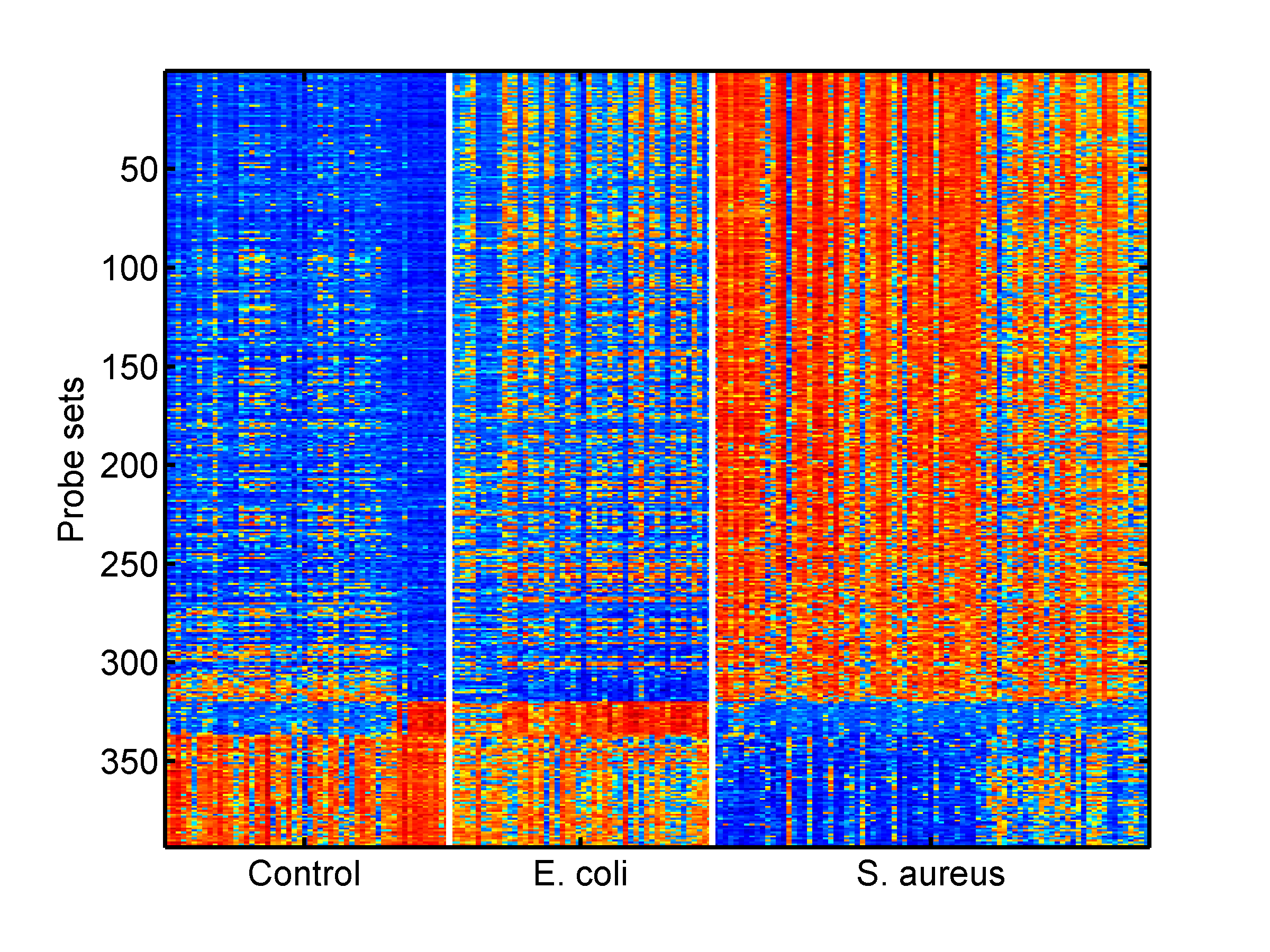


**B**

**A**

Supplement: Figure S2 — Heat maps of genes contributing to the murine S. aureus classifier. (A) Genes within the top five factors contributing to the murine S. aureus classifier were identified and ranked by p-value after Bonferroni correction. A subset of genes (393 after removing duplicates) is depicted here, stratified by pathogen. (B) The same genes depicted in part (A) are categorized first pathogen and then by time since infection. (DOC) [file pone.0048979.s002.doc]

**Figure S3**


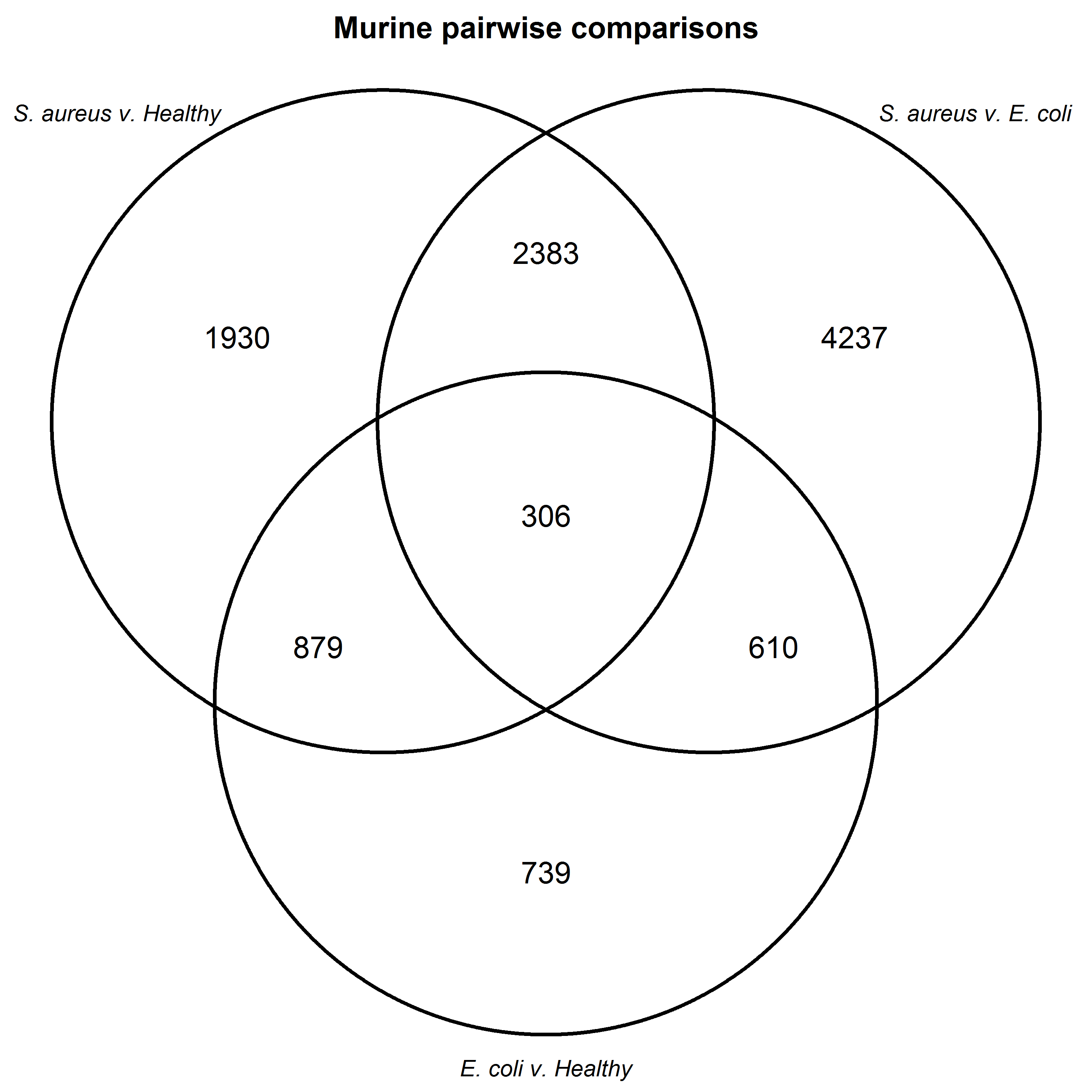

Supplement: Figure S3 — Venn diagram demonstrating the number of overlapping probes in each murine experimental group pairwise comparison. Probes were included that had significantly different levels of expression after Bonferroni correction. (DOC) [file pone.0048979.s003.doc]

**Figure S4**


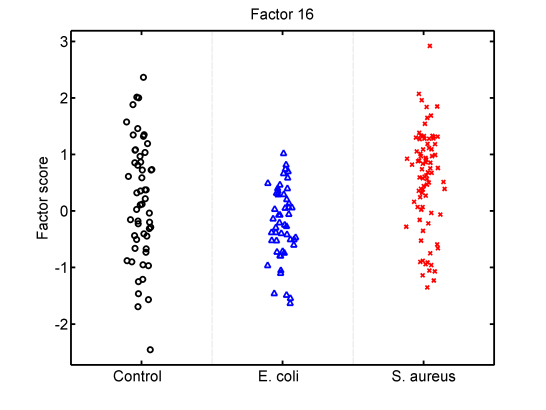

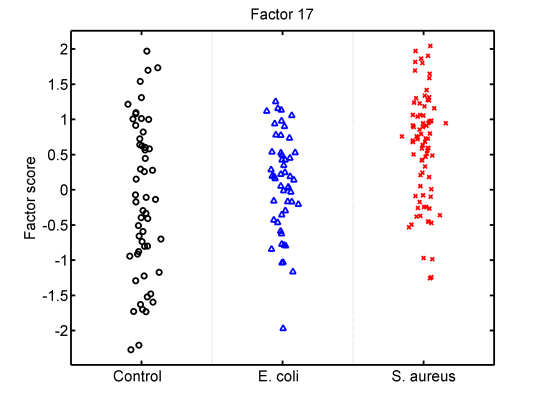

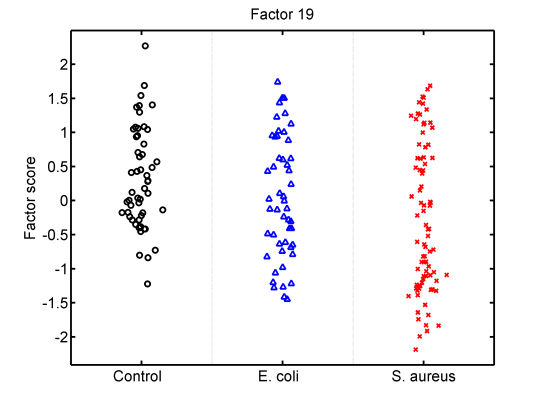

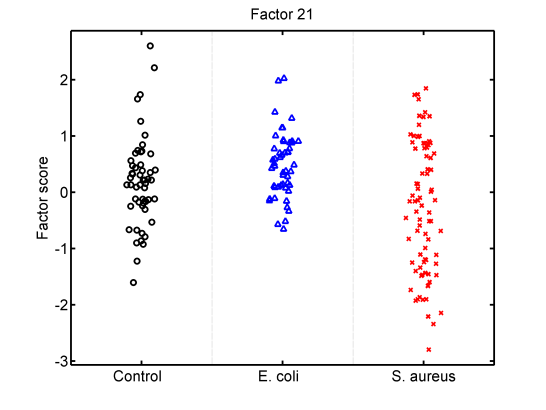

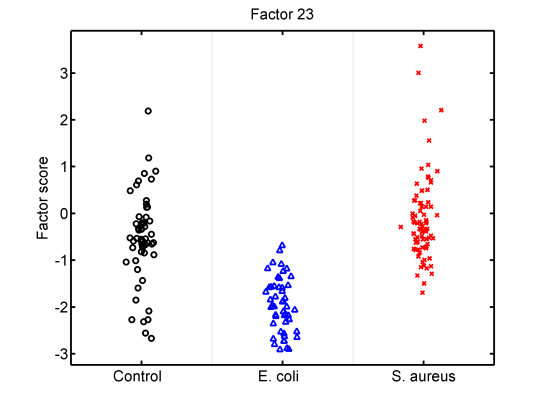

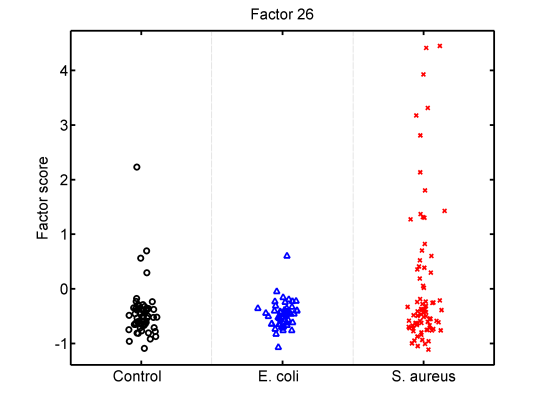

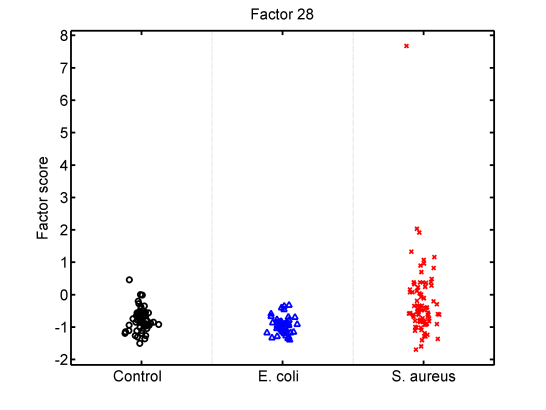

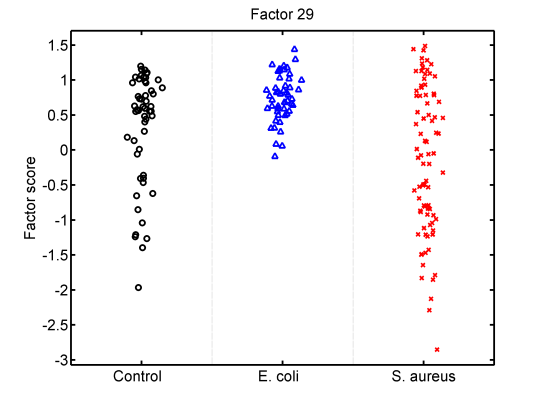

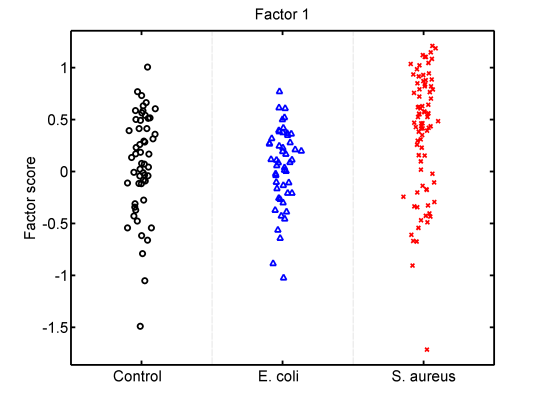

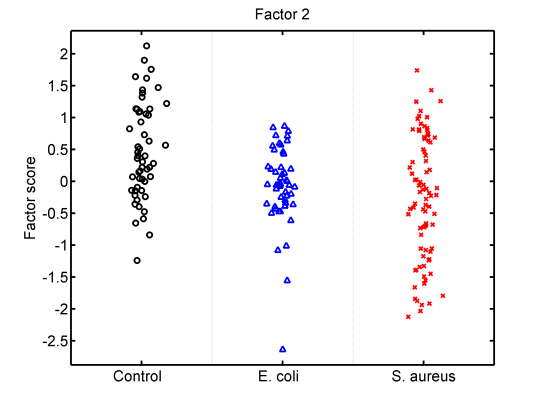

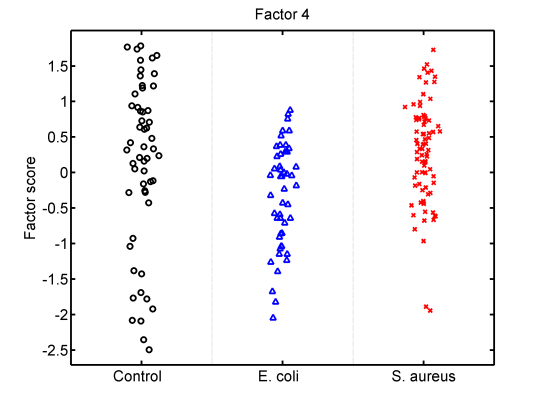

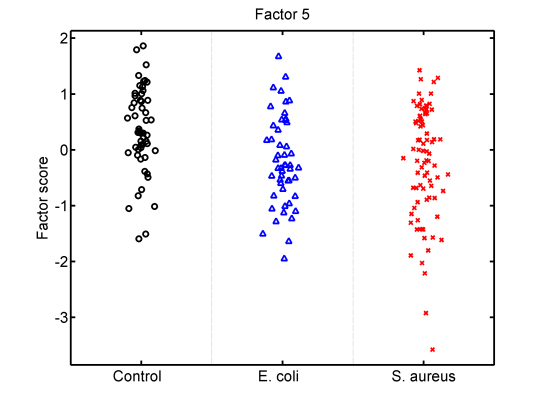

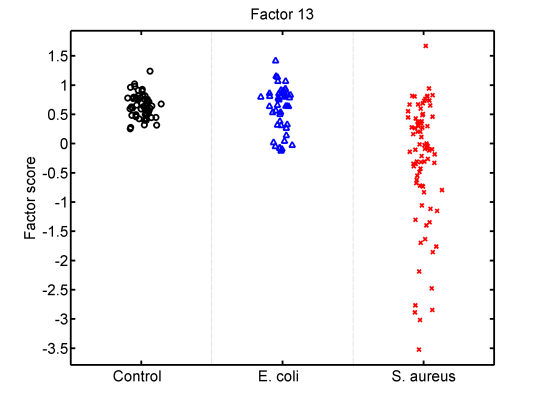

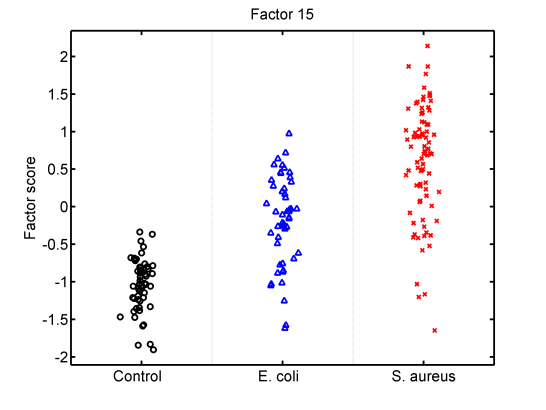

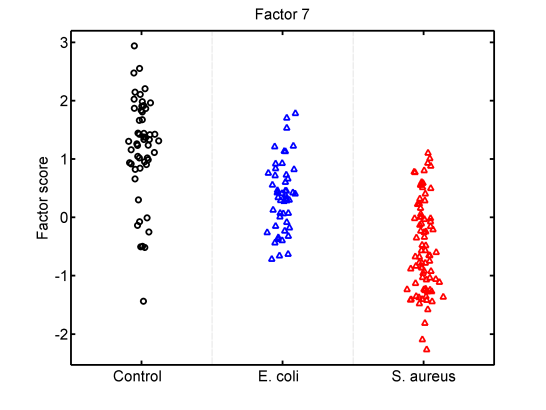

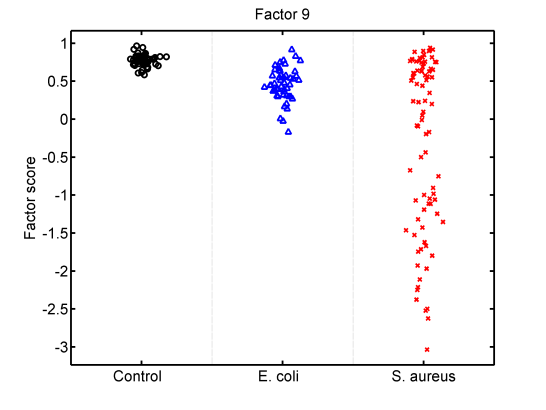

Supplement: Figure S4 — Sixteen murine factors independently associated with S. aureus infection projected onto healthy controls (left panel, black circles), animals with E. coli infection (middle panel, blue triangles), and animals with S. aureus infection (right panel, red “x”). The y-axis represents the factor score. (DOC) [file pone.0048979.s004.doc]

**Figure S5**


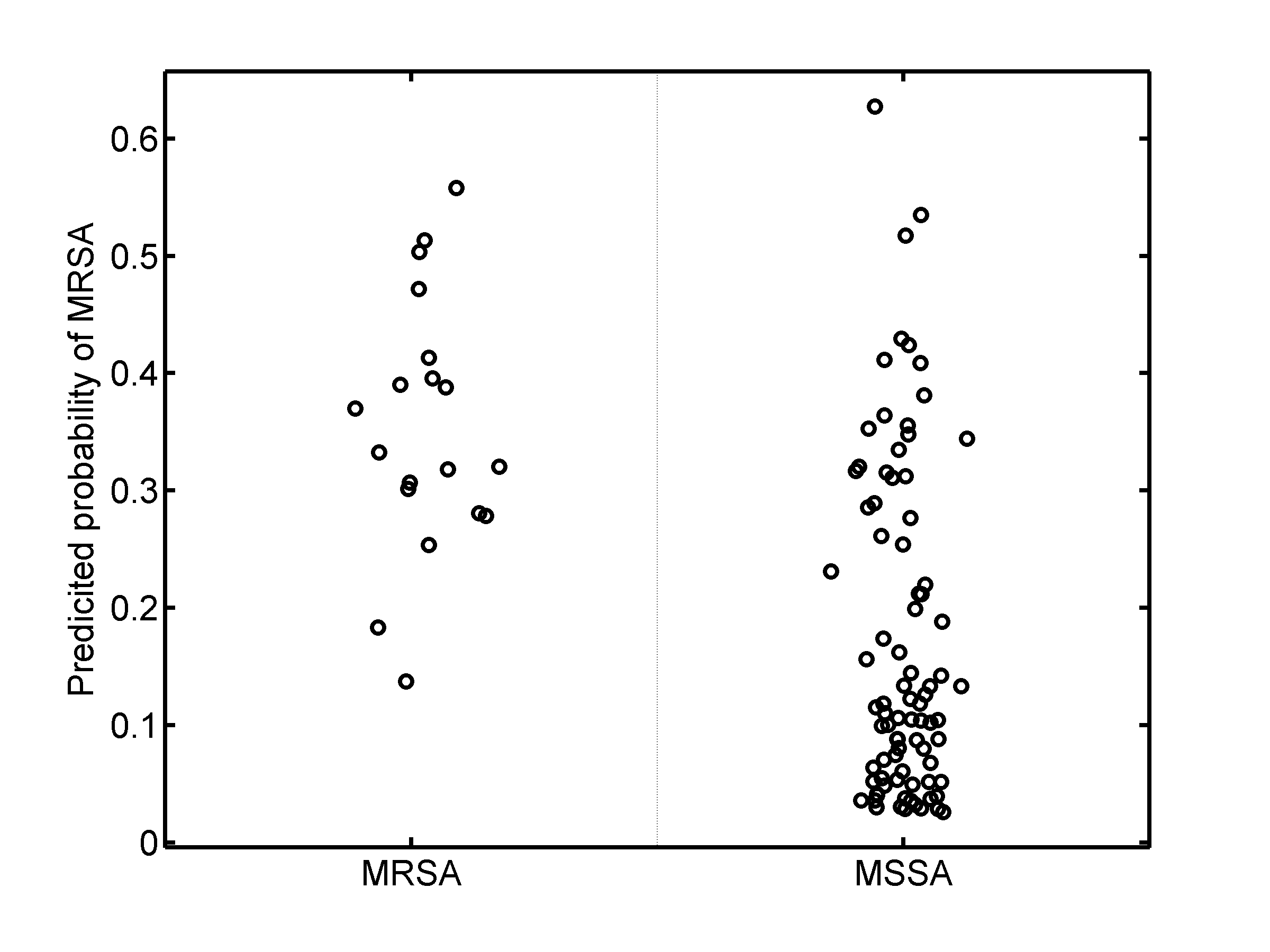

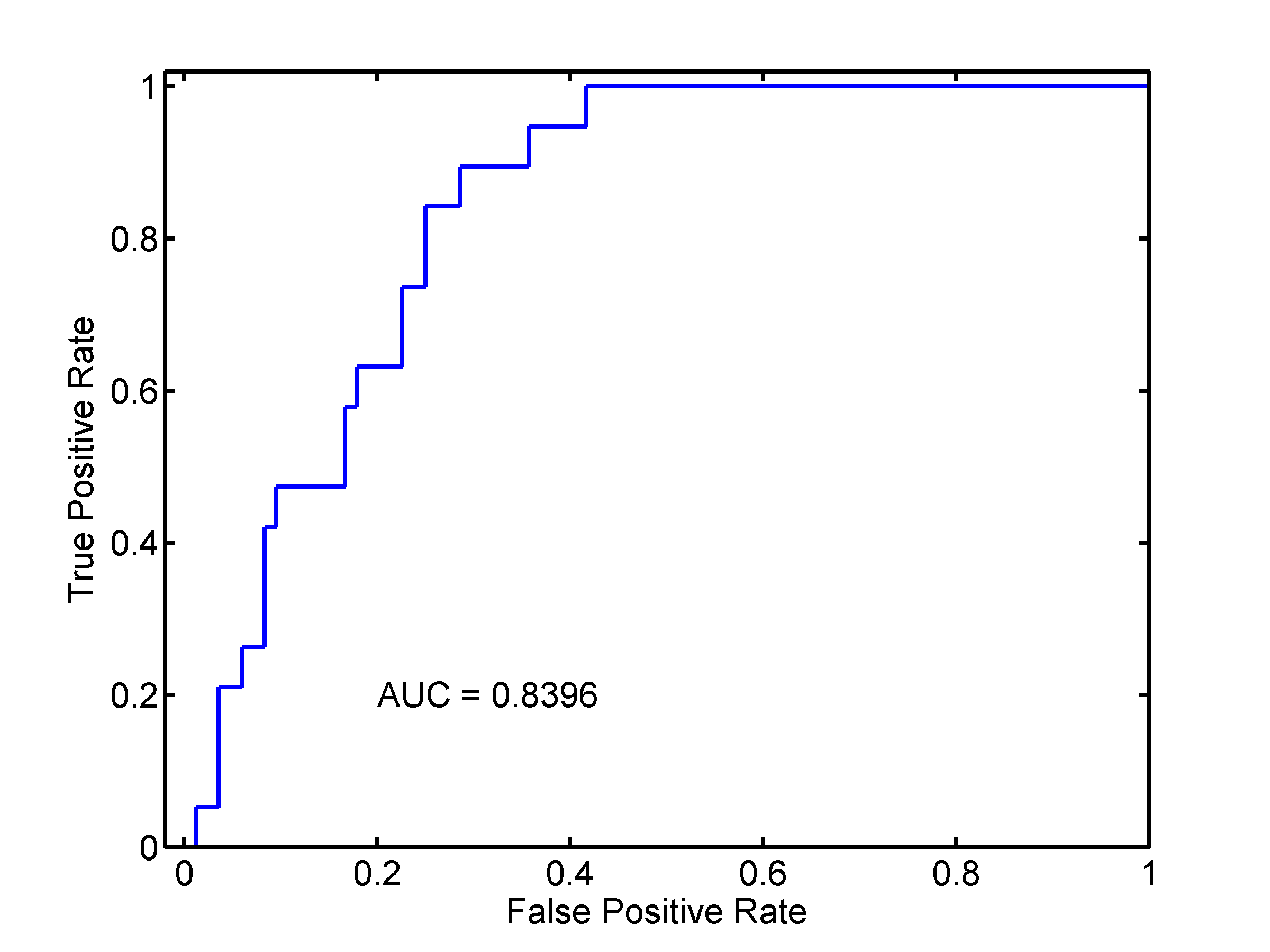

Supplement: Figure S5 — A factor-based classifier distinguishes MRSA from MSSA infection in mice. An ROC curve is shown for this classification. (DOC) [file pone.0048979.s005.doc]

**Figure S6**

**
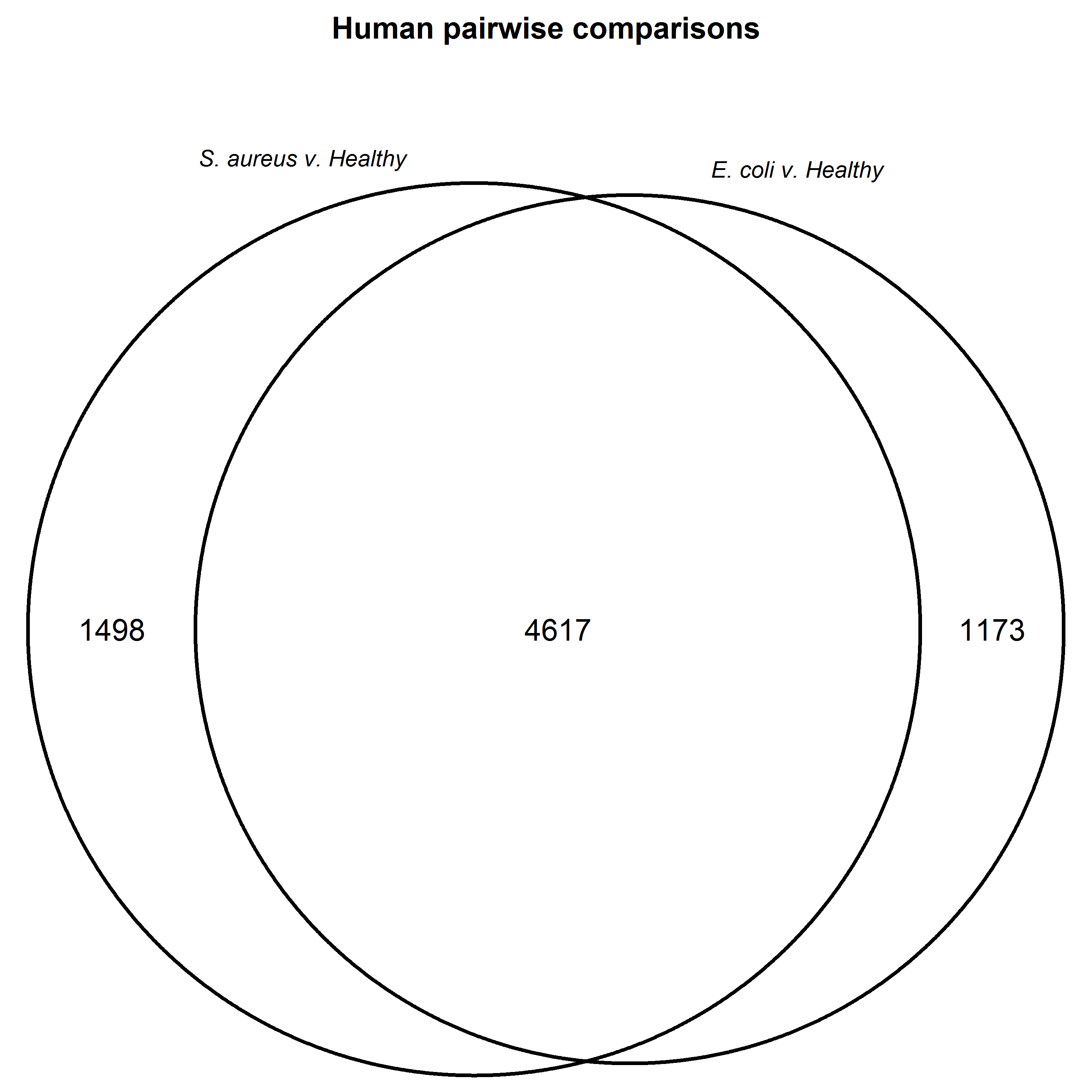
**

Supplement: FIgure S6 — Venn diagram demonstrating the number of overlapping probes in each human experimental group pairwise comparison. Probes were included that had significantly different levels of expression after Bonferroni correction. No probes met this cutoff for the S. aureus vs. E. coli comparison. (DOC) [file pone.0048979.s006.doc]

**Figure S7**


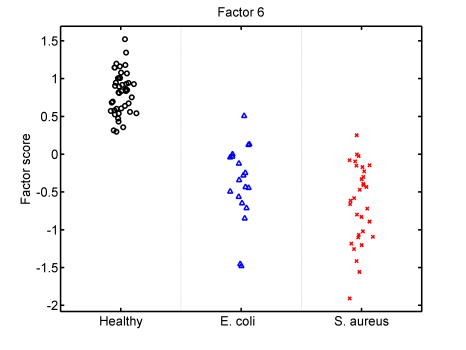

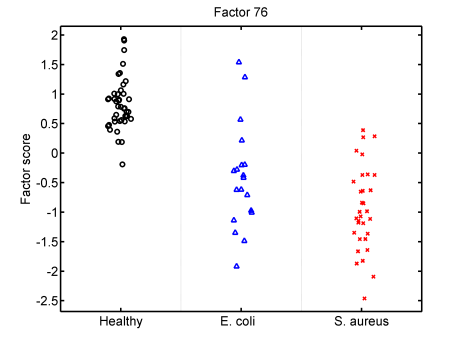

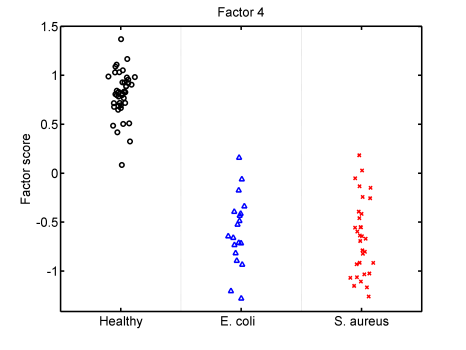

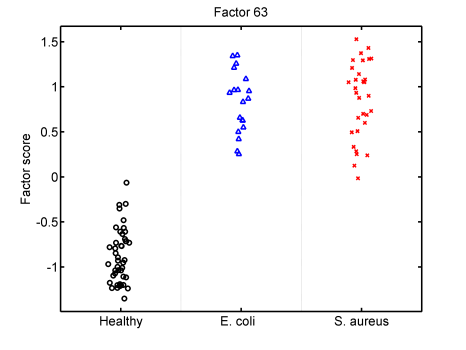

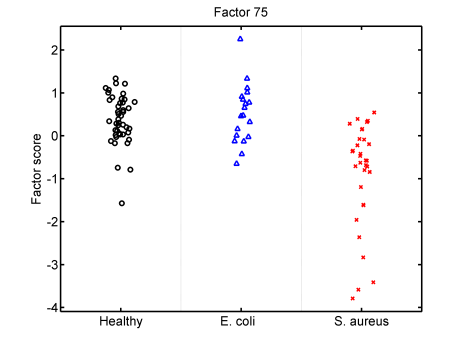

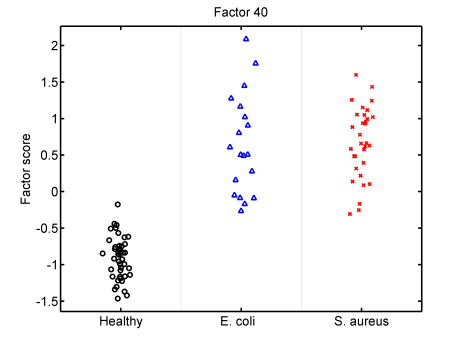

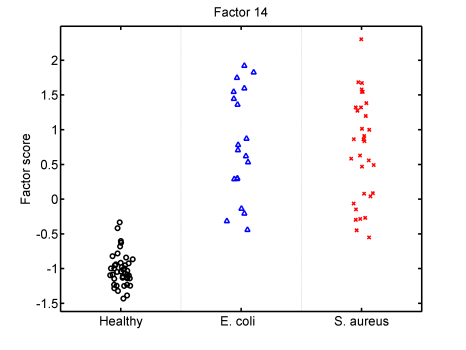

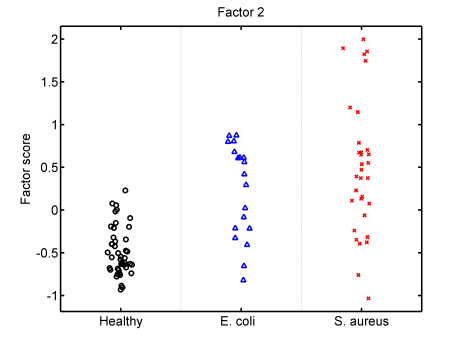

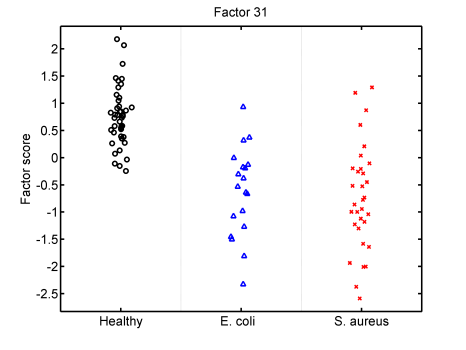

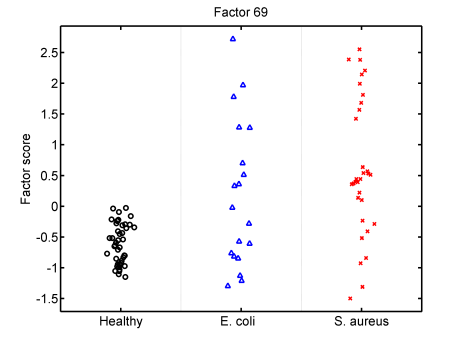

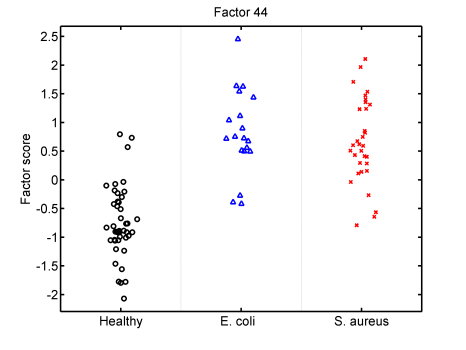

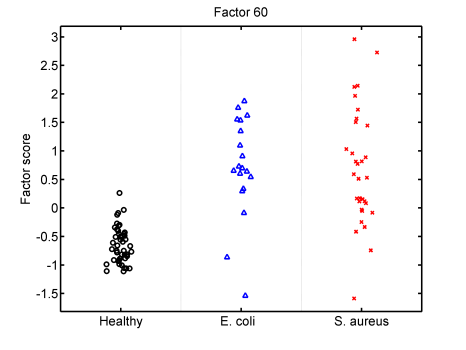

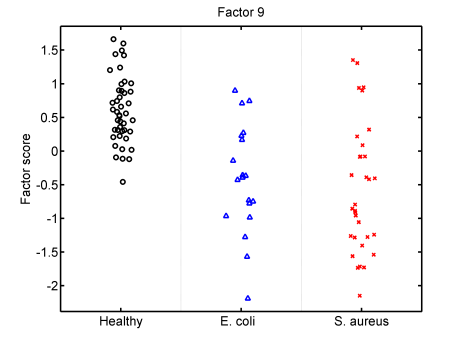

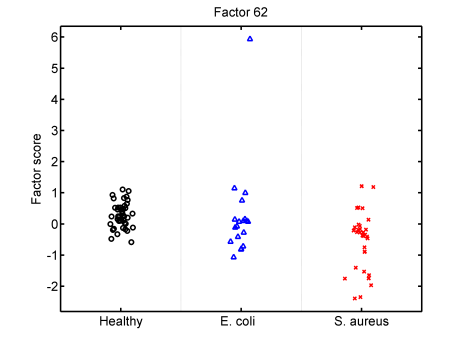

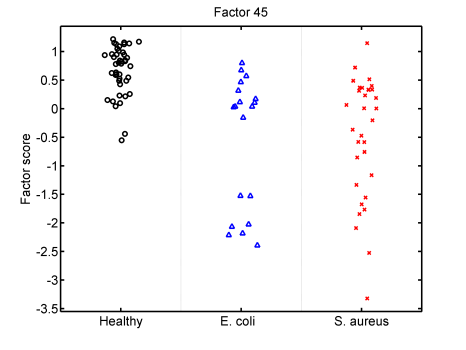

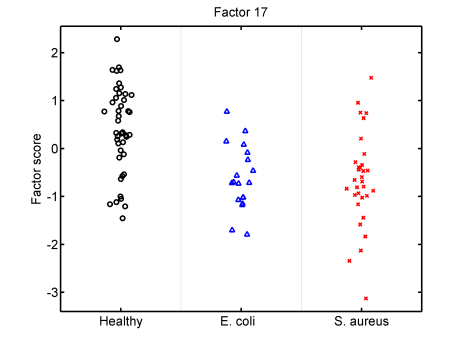

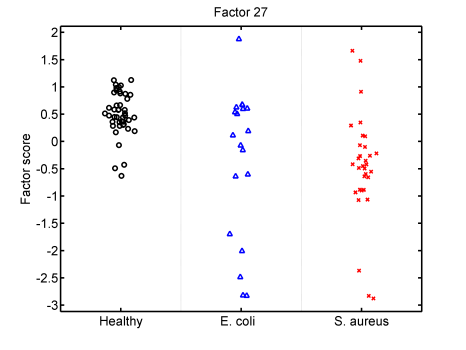

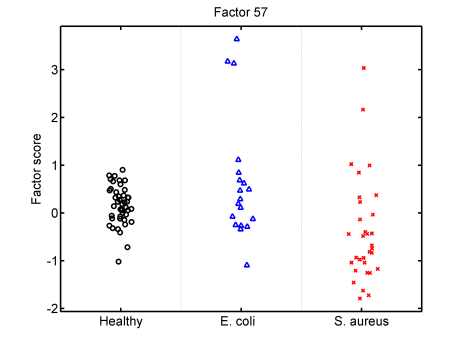

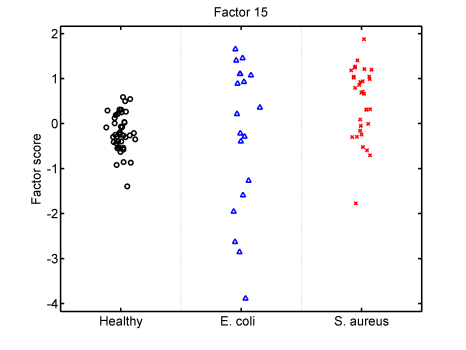

Supplement: Figure S7 — Seventeen human factors independently associated with S. aureus BSI projected onto healthy controls (left panel, black circles), subjects with E. coli BSI (middle panel, blue triangles), and subjects with S. aureus BSI (right panel, red “x”). The y-axis represents the factor score. (DOC) [file pone.0048979.s007.doc]

**Figure S8**


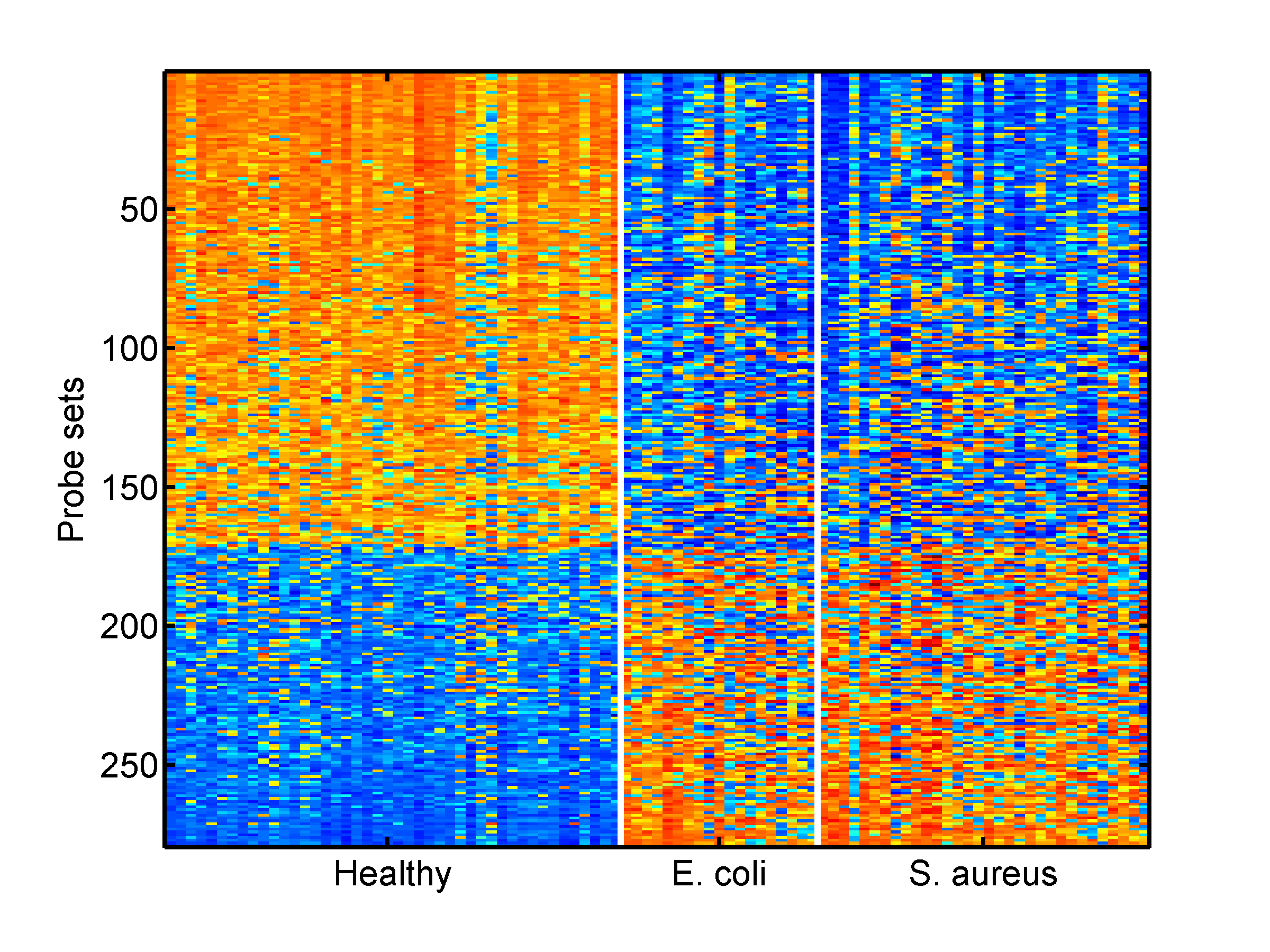

Supplement: Figure S8 — Heat map of genes contributing to the human S. aureus classifier. Genes within the top two factors contributing to the human S. aureus classifier were identified and ranked by p-value after Bonferroni correction. A subset of genes (86 after removing duplicates) is depicted here, stratified by pathogen. (DOC) [file pone.0048979.s008.doc]
