# Supplementary material for: Gene Expression-Based Classifiers Identify Staphylococcus aureus Infection in Mice and Humans
Source: PLoS One. 2013 Jan 9;8(1):e48979. doi: 10.1371/journal.pone.0048979 (PMC3541361; doi:10.1371/journal.pone.0048979)
Supplement: Table S2 — Probes and corresponding genes that were differentially expressed (after Bonferroni correction) in mice with MRSA vs. MSSA infection. (DOC) [file pone.0048979.s010.doc]

**Table S2.** Probes and corresponding genes that are differentially expressed (after Bonferroni correction) in mice with MRSA vs. MSSA infection.

| **Probe Set ID** | **Gene Symbol** | **Gene Title** | **Entrez Gene ID** | **p-value** |
| --- | --- | --- | --- | --- |
| 1420021_s_at | Suz12 | suppressor of zeste 12 homolog (Drosophila) | 52615 | 5.54E-07 |
| 1422842_at | Xrn2 | 5'-3' exoribonuclease 2 | 24128 | 9.49E-07 |
| 1429432_at | Bat2l2 | HLA-B associated transcript 2-like 2 | 226562 | 7.86E-07 |
| 1434391_at | AI503316 | expressed sequence AI503316 | 105860 | 1.02E-06 |
| 1439247_at | Dock10 | dedicator of cytokinesis 10 | 210293 | 1.02E-06 |
| 1444279_at | Huwe1 | HECT, UBA and WWE domain containing 1 | 59026 | 3.22E-07 |
| 1446384_at | --- | (Unannotated) | --- | 1.33E-07 |
| 1446512_at | Zc3h15 | zinc finger CCCH-type containing 15 | 69082 | 2.34E-07 |
| 1449578_at | Supt16h | suppressor of Ty 16 homolog (S. cerevisiae) | 114741 | 2.91E-07 |
| 1450051_at | Atrx | alpha thalassemia/mental retardation syndrome X-linked homolog (human) | 22589 | 6.07E-07 |
| 1451685_at | Mllt6 | myeloid/lymphoid or mixed-lineage leukemia (trithorax homolog, Drosophila); translocated to, 6 | 246198 | 8.09E-07 |
| 1452470_at | Cep350 | centrosomal protein 350 | 74081 | 1.03E-06 |
| 1456112_at | Tpr | translocated promoter region | 108989 | 8.10E-07 |
| 1457731_at | Snapc3 | small nuclear RNA activating complex, polypeptide 3 | 77634 | 3.80E-07 |
| 1459398_at | Peli1 | Pellino 1 | 67245 | 7.47E-07 |
