# Supplementary material for: Gene Expression-Based Classifiers Identify Staphylococcus aureus Infection in Mice and Humans
Source: PLoS One. 2013 Jan 9;8(1):e48979. doi: 10.1371/journal.pone.0048979 (PMC3541361; doi:10.1371/journal.pone.0048979)
Supplement: Methods S1 — A detailed description of microarray preparation. (DOC) [file pone.0048979.s014.doc]

**Methods S1.**

**Microarray Preparation**

Total RNA was extracted using the PAXgene Blood RNA Kit (Qiagen, Valencia, CA) following the manufacturer’s recommended protocol including DNase treatment.  Following isolation, RNA quantity was determined via a Nanodrop UV-Vis Spectrophotometer (Thermo Fisher Scientific, Pittsburgh, PA) and quality via capillary electrophoresis using the Agilent 2100 Bioanalyzer (Agilent, Santa Clara, CA).

Microarray data was collected at Expression Analysis (Durham, NC) using the GeneChip® Human Genome U133A 2.0 Array (Affymetrix, Santa Clara, CA).  Target was prepared and hybridized according to the "Affymetrix Technical Manual". A set of four peptide nuleic acid (PNA) oligomers (Applied Biosystems, Foster City, CA) with sequences complimentary to globin mRNA were added to 2.5 ug of total RNA to reduce globin RNA transcription, then converted into cDNA using Reverse Transcriptase (Invitrogen) and a modified oligo(dT)24 primer that contains T7 promoter sequences (GenSet).   After first strand synthesis, residual RNA was degraded by the addition of RNaseH and a double-stranded cDNA molecule was generated using DNA Polymerase I and DNA Ligase. The cDNA was then purified and concentrated using a phenol:chloroform extraction followed by ethanol precipitation.  The cDNA products were incubated with T7 RNA Polymerase and biotinylated ribonucleotides using an In Vitro Transcription kit (Affymetrix). The resultant cRNA product was purified using an RNeasy column (Qiagen) and quantified with a spectrophotometer. The cRNA target (20ug) was incubated at 94ºC for 35 minutes in fragmentation buffer (Tris, MgOAc, KOAc).  The fragmented cRNA was diluted in hybridization buffer (MES, NaCl, EDTA, Tween 20, Herring Sperm DNA, Acetylated BSA) containing biotin-labeled OligoB2 and Eukaryotic Hybridization Controls (Affymetrix).  The hybridization cocktail was denatured at 99°C for 5 minutes, incubated at 45°C for 5 minutes and then injected into a GeneChip cartridge. The GeneChip array was incubated at 42°C for at least 16 hours in a rotating oven at 60 rpm.  GeneChips were washed with a series of nonstringent (25°C) and stringent (50°C) solutions containing variable amounts of MES, Tween20 and SSPE. The microarrays were then stained with Streptavidin Phycoerythrin and the fluorescent signal was amplified using a biotinylated antibody solution.  Fluorescent images were detected in an GeneChip® Scanner 3000 and expression data was extracted using the GeneChip Operating System v 1.1 (Affymetrix).  All GeneChips were scaled to a median intensity setting of 500.
